# Target site specificity and *in vivo* complexity of the mammalian arginylome

**DOI:** 10.1038/s41598-018-34639-6

**Published:** 2018-11-01

**Authors:** Junling Wang, Vikas Rao Pejaver, Geoffrey P. Dann, Max Y. Wolf, Manolis Kellis, Yun Huang, Benjamin A. Garcia, Predrag Radivojac, Anna Kashina

**Affiliations:** 1 Department of Biomedical Sciences, School of Veterinary Medicine, University of Pennsylvania, Philadelphia, PA 19104 USA; 2 Department of Computer Science, Indiana University, Bloomington, IN 47405 USA; 3 Department of Biochemistry and Biophysics, School of Medicine, University of Pennsylvania, Philadelphia, PA 19104 USA; 4 Broad Institute of MIT and Harvard, and MIT Computer Science and Artificial Intelligence Laboratory, Cambridge, MA USA; 5 Present Address: Department of Biomedical Informatics and Medical Education and the eScience Institute, University of Washington, Washington, USA

## Abstract

Protein arginylation mediated by arginyltransferase ATE1 is a key regulatory process essential for mammalian embryogenesis, cell migration, and protein regulation. Despite decades of studies, very little is known about the specificity of ATE1-mediated target site recognition. Here, we used *in vitro* assays and computational analysis to dissect target site specificity of mouse arginyltransferases and gain insights into the complexity of the mammalian arginylome. We found that the four ATE1 isoforms have different, only partially overlapping target site specificity that includes more variability in the target residues than previously believed. Based on all the available data, we generated an algorithm for identifying potential arginylation consensus motif and used this algorithm for global prediction of proteins arginylated *in vivo* on the N-terminal D and E. Our analysis reveals multiple proteins with potential ATE1 target sites and expand our understanding of the biological complexity of the intracellular arginylome.

## Introduction

Protein arginylation, mediated by the arginyltransferase ATE1, is a posttranslational modification that is essential for mammalian embryogenesis, regulates many fundamental biological processes, and targets a large number of proteins *in vivo*^1^. In mammals, ATE1 is represented by four homologous isoforms ATE1-1, 2, 3, and 4, generated by alternative splicing from a single gene and reported in different studies to have varied activity, substrate specificity, and tissue-specific expression^2–4^. Despite all the accumulating data, the specificity of ATE1 toward different target sites on protein substrates remains a controversial open question in the field.

It has been previously reported that ATE1 adds Arg (R) predominantly to the N-terminally exposed acidic residues, Asp (D) and Glu (E)^5^. It has also been suggested by indirect studies that Cys (C) can undergo arginylation in mammals^6^, and that C arginylation requires its tri-oxidation to cysteic acid – a mechanism that has been proposed to mediate intracellular oxygen sensing in mammalian systems^7^ and in plants^8^. At the same time, mass spectrometry analysis has identified a number of peptides in native protein samples that are arginylated on other N-terminally exposed residues^9^. In these tests, most amino acids were found arginylated, including unmodified C, even though the relative amount of this arginylation, compared to the conventional D/E, has never been estimated. Arginylation of non-acidic residues has never been systematically tested *in vivo* or in ATE1-mediated assays *in vitro*.

A few years ago, our group discovered that in addition to N-terminal arginylation, ATE1 can also add arginine to the acidic side chains of D and E on the mid-chain sites of intact proteins^10^. Such arginylation was also observed or implicated in other studies^11,12^. Surprisingly, this reaction, while mediated by the same enzyme, results in a different arginine linkage to the protein substrate – via an isopeptide bond between the arginine amino group and the side chain carboxyl group of the target site, rather than via a conventional peptide bond at the protein’s N-terminus. This discovery further expands the potential biological scope of arginylation, since arginylation at midchain sites can in principle occur on any proteins *in vivo* without a requirement for prior priming by proteases or endopeptidases, similar to other major regulatory posttranslational modifications.

Despite decades of study, many open questions remain about the target site specificity of ATE1 and the possibility that different ATE1 isoforms may mediate different types of arginylation. This topic remains a focus of debate in the literature^13^. Here we use peptide arrays, *in vitro* assays, and computational analysis to dissect the target site specificity of different ATE1 isoforms and to estimate the complexity of the intracellular arginylome. Our results support the existence of “non-canonical” ATE1-mediated arginylation, provide new tools for arginylation prediction, and point to hundreds of proteins that can be explored as potential novel targets of arginylation *in vivo*.

## Results

### Design of the peptide array for dissecting target site specificity of ATE1

To dissect target site specificity of ATE1, we utilized the SPOT synthesis peptide array to generate oriented sets of peptide substrates immobilized on a cellulose membrane, which could be utilized in the arginylation reaction. We based the design of these peptides on a model peptide substrate, DIAALVHSSGMC, previously designed by us based on the sequence similarity with the N-terminus of non-muscle beta actin, and proven in our prior studies to be a favorable substrate for all four ATE1 isoforms^10,14^. This peptide has been previously shown to be arginylated *in vitro* on the N-terminal aspartate (D), and it has no other sites within the sequence that could in principle be arginylated when immobilized on the array. We also produced an equivalent of this peptide with the N-terminal glutamate (EIAALVHSSGMC). These two peptides series were synthesized on the array (array 1) with the following variations.

First, to test for potential site preference for mid-chain D/E arginylation, we varied the position of the target D or E within the sequence, from position 2 to the end (Fig. S1, left column; the target residues are shown in bold underlined font). In these peptides, we designed the N-terminal residue to be S, based on a peptide we previously tested *in vitro* and found to be arginylated on the side chain of an internal D^10^.

Second, to test for the specificity of ATE1 isoforms toward the N-terminal residue, we varied the identity of the first residue in the peptide, using all 20 amino acids as the possibilities (Fig. S1, middle column).

Finally, to complete the array, we added several natural peptides we have previously identified by mass spectrometry in various biological samples^9^. In these peptides, the arginylated residue was identified at the N-terminus by mass spectrometry, but we considered the possibility that the preferred target residue in these peptides could also fall on the nearest mid-chain D or E (as denoted in bold underlined font in the left column in Fig. S1).

Each pattern was repeated on the array 8 times, enabling us to analyze 4 mouse ATE1 isoforms in duplicates on the same membrane. The last three positions in each array were left blank, to serve as negative controls.

### Mouse ATE1 isoforms have different substrate specificity, broader than previously believed

**Figure 1:**
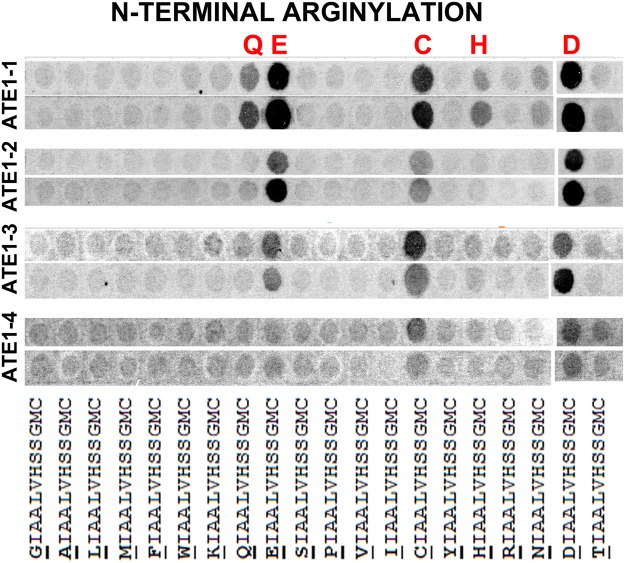
Four mouse ATE1 isoforms have different, partially overlapping specificity toward their N-terminal target sites. Cropped image of the autoradiogram showing Arg incorporation as the ^14^C signal (gray or black after the film exposure). ATE1 isoforms are indicated on the left. In each ATE1 isoform group, two rows show the two repeats of the same experiment, performed using the arrays synthesized in duplicates on the same membrane and analyzed in duplicates in the arginylation assays (see Fig. S1 for the original uncropped image). The peptide sequences are shown underneath, with the target residue underlined. The peptide backbone was selected based on the N-terminal beta actin peptide, as explained in the text.

**Figure 2:**
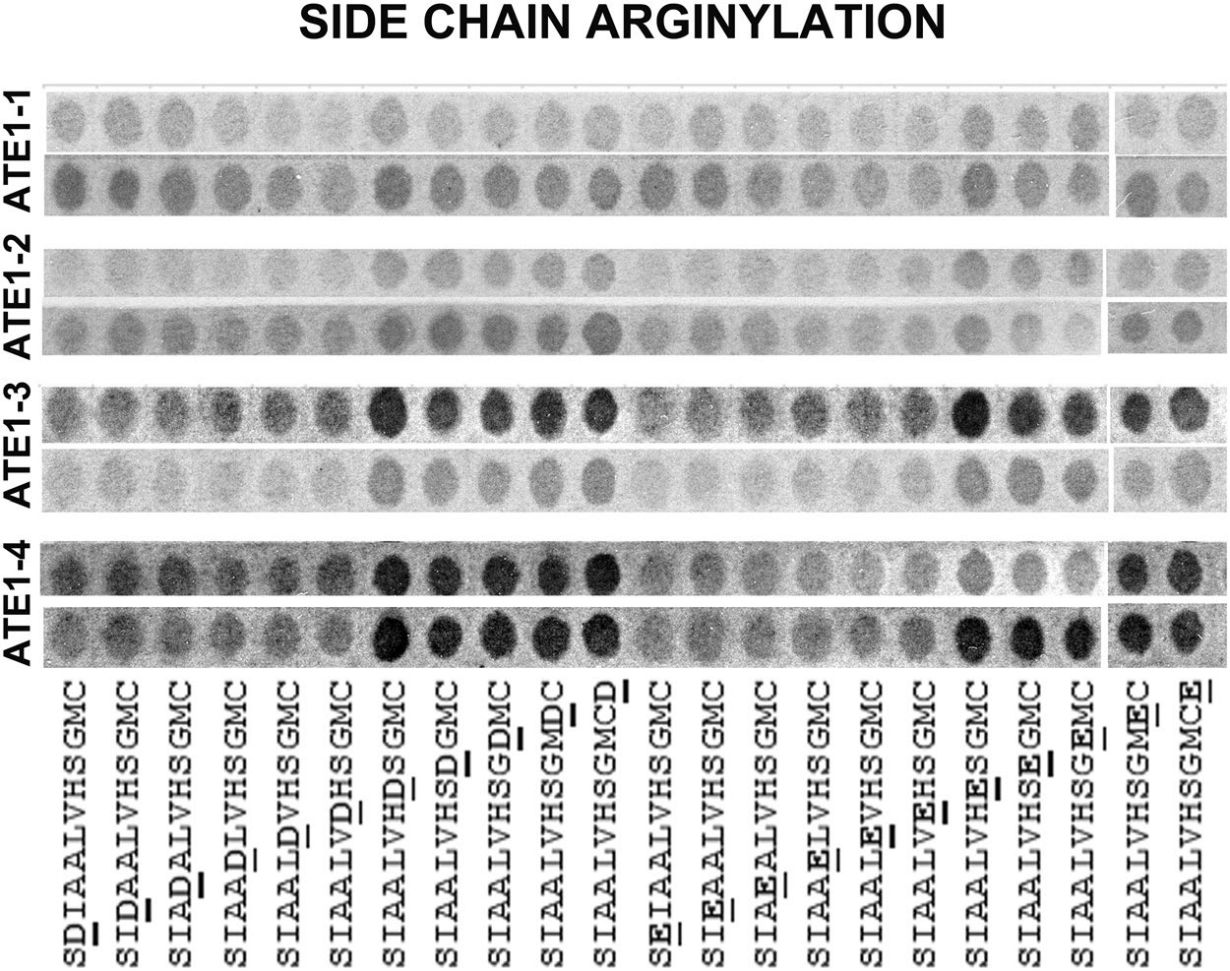
Four mouse ATE1 isoforms have different specificity toward midchain target sites. Cropped image of the autoradiogram showing Arg incorporation as the ^14^C signal (gray or black after the film exposure). ATE1 isoforms are indicated on the right, two rows show the two repeats of the same experiment, performed using the arrays synthesized in duplicates on the same membrane and analyzed in duplicates in the arginylation assays (see Fig. S1 for the original uncropped image). The peptide sequences are shown underneath, with the target residue underlined.

**Figure 3:**
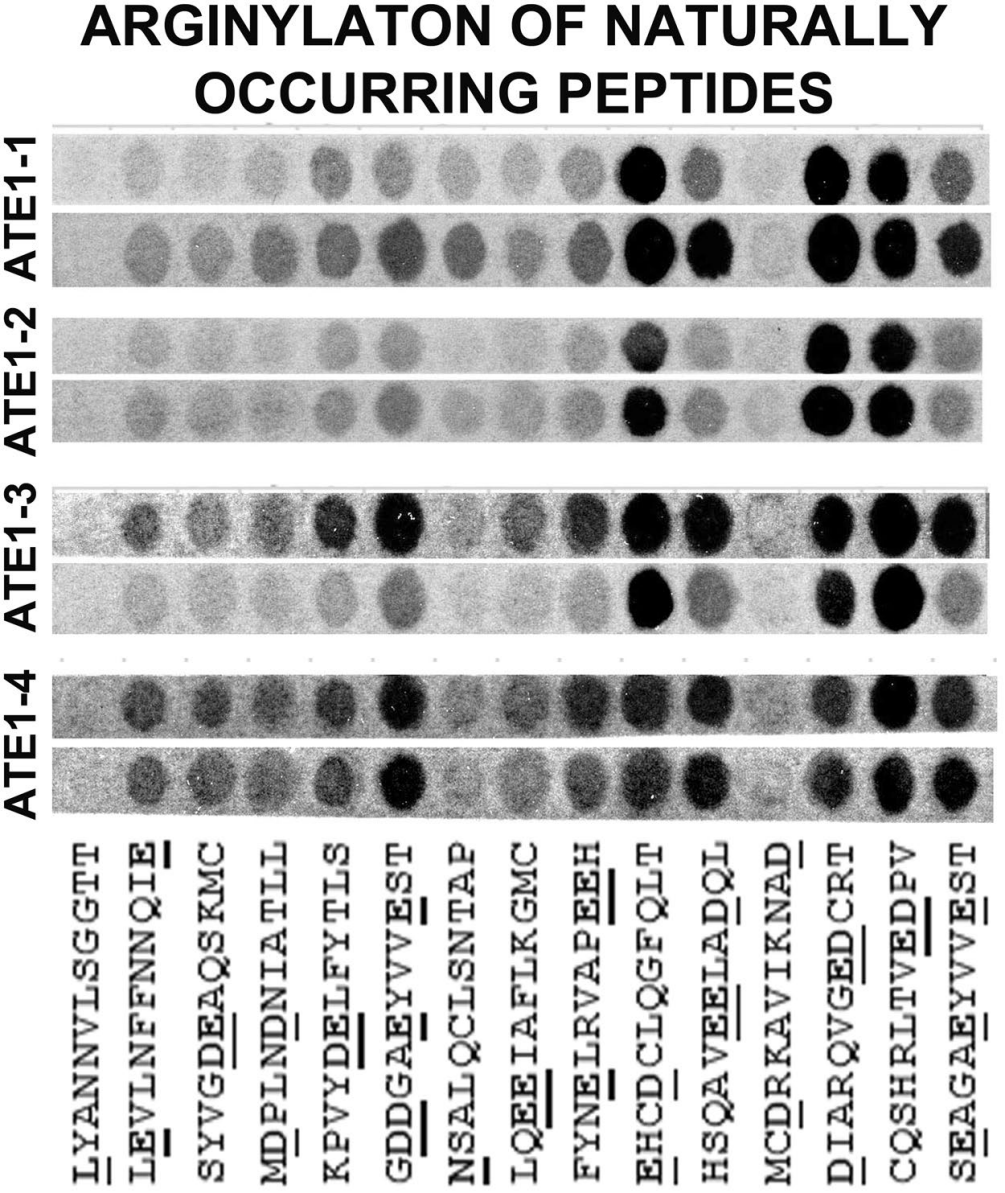
Four mouse ATE1 isoforms show different reactivity with natural peptides. Cropped image of the autoradiogram showing Arg incorporation as the ^14^C signal (gray or black after the film exposure). ATE1 isoforms are indicated on the right, two rows show the two repeats of the same experiment, performed using the arrays synthesized in duplicates on the same membrane and analyzed in duplicates in the arginylation assays (see Fig. S1 for the original uncropped image). The peptide sequences are shown underneath, with the target residue underlined.

To test the ability of each of the four ATE1 isoforms to arginylate the arrayed peptides, we incubated each membrane in the reaction mix for *in vitro* peptide arginylation^10,14^, containing ATE1, R-tRNA synthetase, R-specific tRNA, and [^14^C-R] as a free amino acid. We then washed out the reaction components (including RNase treatment to destroy the residual 14C-R-tRNA) and developed the membrane using X-ray film to detect the ATE1-dependent incorporation of [^14^C-R] into the immobilized peptides (see Fig. S2 for the raw data of the exposed X-ray film, from which the data was cropped and shown in the main text Figs 1–3).

Remarkably, the four mouse ATE1 isoforms showed prominent and consistent differences in target site specificity, both at the N-terminus and the side chain sites (Figs 1–3). At the N-terminal sites (Fig. 1), only three of the four ATE1 isoforms (ATE1-1, 2, and 3) showed high preference for the peptides containing N-terminal D and E. ATE1-4 did not appear to target peptides containing N-terminal E. At the same time all four isoforms, to a various degree, showed prominent reactivity with the peptides bearing N-terminal C. Even more strikingly, ATE1-1, unlike any other ATE1 isoforms, appeared to be reactive with additional N-terminal sites not seen with other ATE1 isoforms, including Q and, weakly, H. Thus, it appears that N-terminal target site specificity of ATE1-1 may be broader than other ATE1 isoforms and potentially include non-canonical N-terminal residues.

The four ATE1 isoforms also showed different reactivity with the peptides bearing side chain target sites (Fig. 2). In the case of ATE1-1 and ATE1-2, the signal with these peptides containing side chain target sites was substantially lower or absent compared to the peptides containing favorable N-terminal target sites. In our previous studies we observed side chain arginylation of one of these peptides with ATE1-2 in solution^10^. Thus, it appears likely that the peptide array format is unfavorable for side chain targeting by these ATE1 isoforms – possibly due to the absence of the essential secondary structure, or other issues with the accessibility of the peptide. However, we detected substantially higher reactivity of side chain peptide targets with ATE1-3, and especially ATE1-4. This reactivity appeared to be higher in the case of the mid-chain D and E flanked by polar residues (the last 5 peptides in each set), and absent or very weak in the case of the non-polar ones (the first six peptides in each set), consistently for both D and E-containing peptides. Thus, it appears likely that ATE1-3 and ATE1-4 work better with side chain targets in this assay.

All four ATE1 isoforms showed a largely similar specificity toward the naturally occurring peptides placed into the array (Fig. 3), with slight variations that might reflect different isoforms’ preference for a different sequence context. In each of these peptides, multiple sites at the N-terminus and side chain can theoretically be targeted by arginylation (underlined in Fig. S1 and Fig. 3). It appears possible that the four ATE1 isoforms may actually be targeting different sites on these peptides.

### ATE1-1-dependent arginylation of “non-canonical” residues

**Figure 4:**
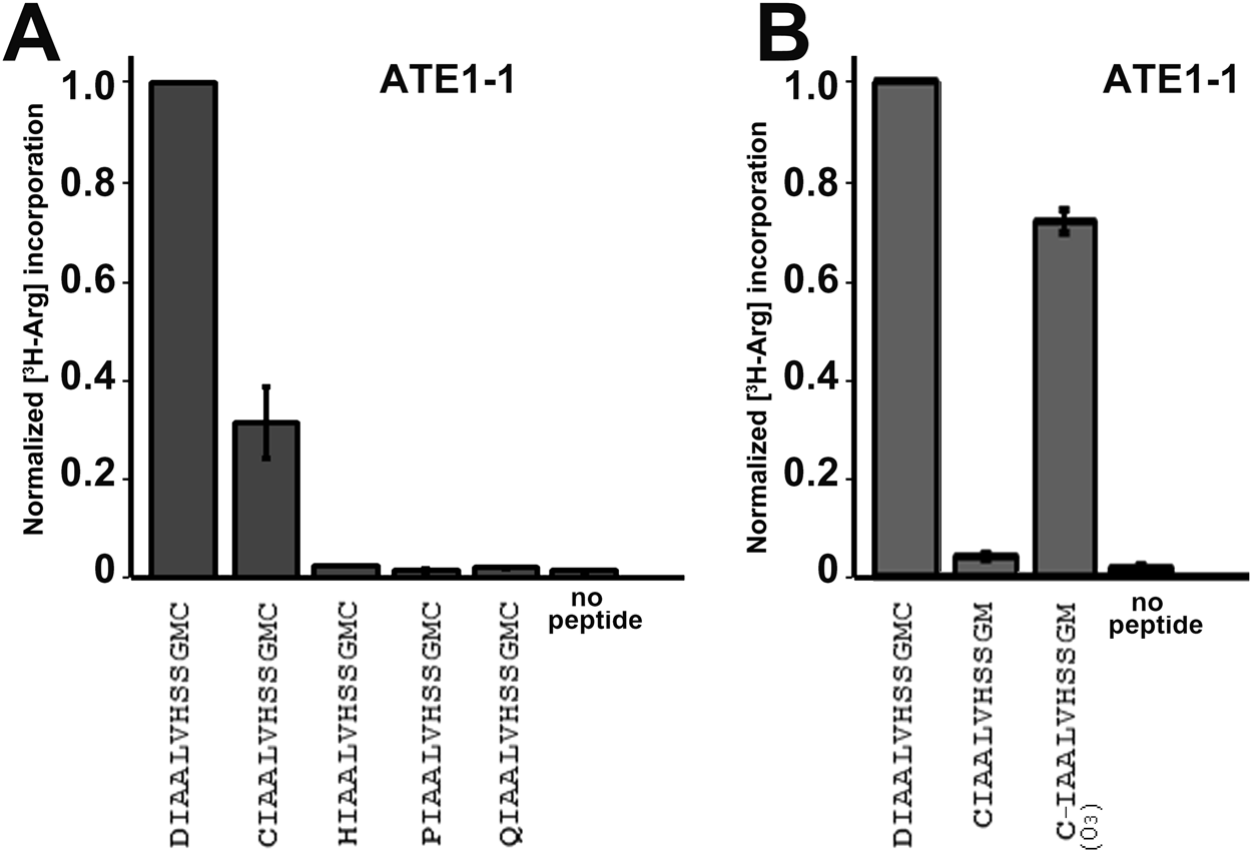
Arginylation of “non-canonical” N-terminal residues. (**A**) Arginylation assay with synthesized peptides in solution with varied residues in the N-terminal position (sequences are indicated on the x axis). Y axis shows [^3^H-R] incorporation signal, normalized to that of the D-containing peptide. Error bars represent SEM, n = 3 independent repeats. See Dataset 1 for the mass spectra of the D- and C-peptides. (**B**) Arginylation assay with the peptide containing N-terminal D (as a positive control) as well as unoxidized and oxidized C (sequences are indicated on the x axis; oxidation is denoted as (O_3_). Error bars represent SEM, n = 3 independent repeats.

To further test the ability of purified ATE1-1 to mediate arginylation of “non-canonical” (i.e., not D or E) residues in an *in vitro* reaction, we synthesized some of the peptides with the N-terminal residues that showed exclusive N-terminal reactivity with ATE1-1 (Q and H), as well as C (whose ability to be directly arginylated without oxidation has generated some past controversy^13^) to test their arginylation in solution rather than in the array format. We also included peptides containing N-terminal D and P (as a positive and negative control, respectively). We used these peptides to perform *in vitro* arginylation assays in solution, followed by peptide isolation on a reverse phase column, acetonitrile elution, and scintillation counting as described in^10^ to determine [^3^H-R] incorporation (Fig. 4A). In this assay, the peptide containing N-terminal C showed noticeable incorporation of the radioactive label, consistent with the peptide array data and suggesting that N-terminal C can serve as a direct ATE1 target. No significant increase in R incorporation over the negative control was observed using peptides with N-terminal Q and H, suggesting differences between the array format and the in-solution peptide arginylation assay. Thus, our present data cannot fully support or disprove the possibility of Q and H arginylation.

To test whether the C-containing peptide is arginylated directly or after tri-oxidation to cysteic acid, we performed arginylation reaction on this peptide using heavy isotope-labeled R [^13^C^15^N-R], and analyzed this arginylated peptide using LC MS/MS. In this analysis we found peptide species containing both tri-oxidized and non-oxidized N-terminal C (Dataset 1). Both peptide species were found arginylated on the N-terminus (Dataset 1). To compare the relative efficiency of unoxidated and tri-oxidated C, we synthesized the peptide containing N-terminal fully unoxidized C (kept in anaerobic conditions during synthesis and storage up to the time just prior to the reaction), as well as the peptide with the same amino acid sequence with fully trioxidated N-terminal C (incorporated into the peptide as the cysteic acid), and compared the incorporation of [^3^H-R] into these peptides by scintillation counting (Fig. 4B). Oxidated C was arginylated to the levels comparable to the positive control (the peptide with N-terminal D), while unoxidated C arginylation was virtually undetectable. Thus, consistent with the published studies, Cys trioxidation greatly facilitates its arginylation.

### Analysis and prediction of the sequence context around the arginylation site

**Figure 5:**
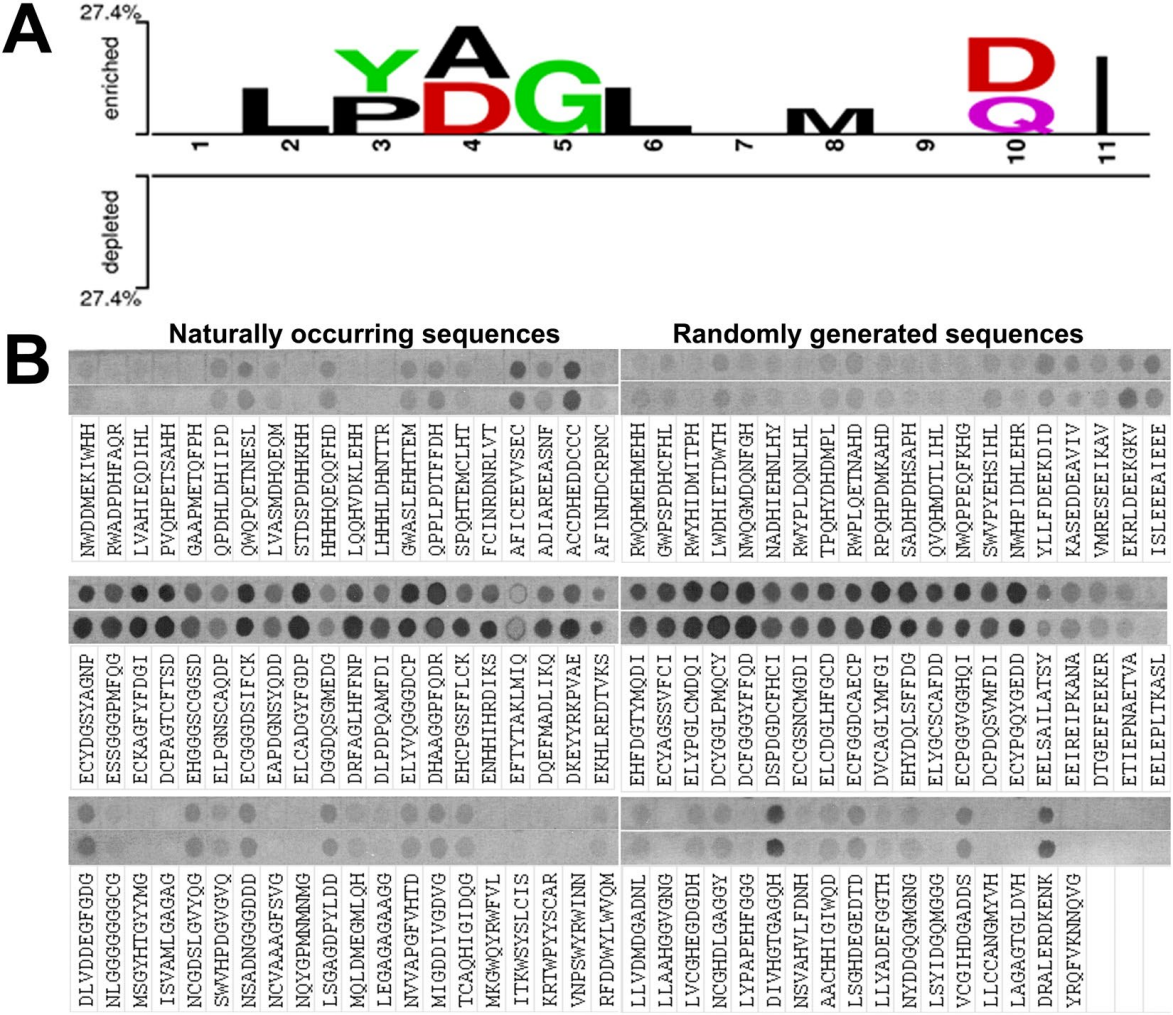
Mouse ATE1-1 exhibits complex specificity toward randomly selected peptide targets. (**A**) Two-sample logo of the arginylated peptides in the literature set against the (restricted) unlabeled peptides. Two Sample Logo was run with default parameters and color settings (no Bonferroni correction was applied). (**B**) Cropped image of the autoradiogram showing R incorporation as the ^14^C signal (gray or black after the film exposure), using peptides with potential side chain D/E target sites (top row), N-terminal D/E target sites with different context in the adjoining residues (middle row), and randomly designed peptides with variable target sites immobilize on the same membrane (bottom row) performed in two independent repeats (see Fig. S3 for the original uncropped image). The peptide sequences are shown underneath. The last three positions in the array were left empty for the negative control.

To further analyze the sequence context favorable for arginylation, we applied a set of predictions to generate targeted peptide arrays, with the eventual goal of developing an algorithm that could predict proteins arginylated *in vivo*. We based these predictions on the sequences of all of our previously published arginylated peptides identified during our analysis of *in vivo* samples^9,15–17^ and synthesized the arrays to contain both the predicted favorable and unfavorable arginylation targets, derived from both naturally occurring and randomly predicted sequences that included D/E as a mid-chain or N-terminal target site, as well as sequences without an imposed requirement on the identity or position of the target residue. Sequences from mouse and human genomes were used for these predictions, utilizing a baseline algorithm that relied on an assumption of the existence of the arginylation consensus sequence (compiled based on the analysis of all the identified arginylation sites, Fig. 5A).

These predicted peptides, when arginylated on the array (referred to as array 2), showed consistently positive signal if they had N-terminal D/E, but not in the case of other N-terminal residues or D/E in the midchain positions, suggesting that midchain arginylation cannot be reliably tested by this assay (Fig. 5B). Moreover, the positive arginylation signal on these peptides did not show a strong correlation with our predicted scores for these peptides in the case of naturally occurring sequences, even if a mild correlation was seen in the case of randomly generated sequences (the last five positions on the right-hand panel of the middle row in Fig. 5B). This result suggests that consensus sequence may not be a good predictor for arginylation. Overall, it appeared that arginylation may depend on the general physicochemical environment around the D or E residue (e.g. hydrophobicity or net charge), rather than a bona fide consensus sequence.

**Figure 6:**
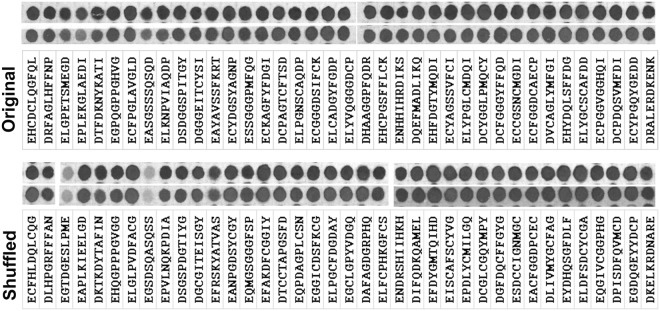
Arginylation of N-terminal D or E occurs independently of the specific order of amnino acid residues around the target site. Cropped image of the autoradiogram showing R incorporation as the ^14^C signal (gray or black after the film exposure), using pairs of peptides with the same amino acid sequence, synthesized in the original (top row) and shuffled (bottom row) order of the residues. In all cases except two, shuffling did not affect the arginylation signal on the peptide. Two repeats are shown for each reaction.

To test this further, we focused on N-terminal arginylation and generated a new prediction, designing pairs of peptides containing the same amino acid residues, but with their order shuffled between the two peptides (“original” and “shuffled” in Fig. 6). This prediction was based on our previously designed tests of the well-known S-palmitoylated peptides and their shuffled versions that tested for positional dependence of flanking residues^18^. We applied only two constraints to this design: (1) the first residue of each peptide was set as D or E and excluded from the random shuffling process, and (2) the shuffled version was set to be highly dissimilar from the original peptide. Guided by these criteria, we synthesized an array with 38 pairs (original-shuffled) of peptides and investigated whether the shuffled peptides were arginylated *in vitro* at the same rate as the original peptides. We used ATE1-1 to perform arginylation on these arrays.

Arginylation was observed all but two of the 38 shuffled peptides (Fig. 6), demonstrating that randomly shuffled peptides with D/E at the N-terminus performed largely similarly to the original in the arginylation assays. Thus, favorable arginylation likely depends only on the presence of certain residues around the arginylation site, but not on their exact order in the sequence.

In order to assess whether features reflective of the local environment around an arginylation site help improve its prediction, we constructed a more sophisticated predictor that incorporated 294 features, including a number of criteria for the peptide sequence such as amino acid composition, net and total charge and hydrophobicity, and other peptide properties, into a logistic regression ensemble predictor. For the purpose of comparison, this predictor was trained on arginylated peptides from the literature and peptide array sets, as well as the restricted unlabeled set derived from the 34 arginylated proteins in our previously designed positive set (Table S1). In 10-fold cross-validation experiments, the area under the ROC curve (AUC) scores of the logistic regression ensemble and the motif-based predictor were found to be 0.74 and 0.68, respectively. Interestingly, when these predictors were trained without the peptides from array 2, the AUCs dropped to 0.63 (logistic regression ensemble) and 0.53 (motif-based), suggesting that the addition of the array 2 data set also contributed to the improved performance. We note that although these increases in AUC can be attributed to the choice of data set, model and the features included, it may have also been a result of overfitting due to the training of a complex model using a small data set.

Based on this algorithm, we constructed another data set of 222 peptides, each containing only one occurrence of D or E (first position), to ensure that the arginylation site could be localized correctly (see Fig. S4 for the peptide sequences and arginylation assay output). This yielded a large data set that was characteristically different from the above data set and thus enabled us to finalize our algorithm.

### Identification of the arginylation-favorable sequence motif

To estimate how many proteins *in vivo* contain sequences predicted by our algorithm to be favorable for arginylation and assess predictive performance, we retrained our logistic regression ensemble predictor using a positive set and the true unlabeled set. This modified version performed comparably to the model trained on the restricted unlabeled set and was constructed mainly for practical application purposes (AUC = 0.73). We then ran this predictor on the human and mouse proteomes, and selected low-, medium- and high-scoring peptides randomly for experimental validation. The largest fraction of arginylated peptides was observed in the medium-scoring sets for both human and mouse (Table S2). However, the fractions of arginylated peptides in the low- and high-scoring sets were comparable in both species, indicative of a high false-negative rate for the predictor. This can be explained by the fact the predictor was trained on arginylated peptides that mostly contained multiple instances of D and E residues and perhaps relied too much on this feature. We concluded that even the combined set of literature- and array-derived peptides was too small to learn a generalizable predictive model.

**Figure 7:**
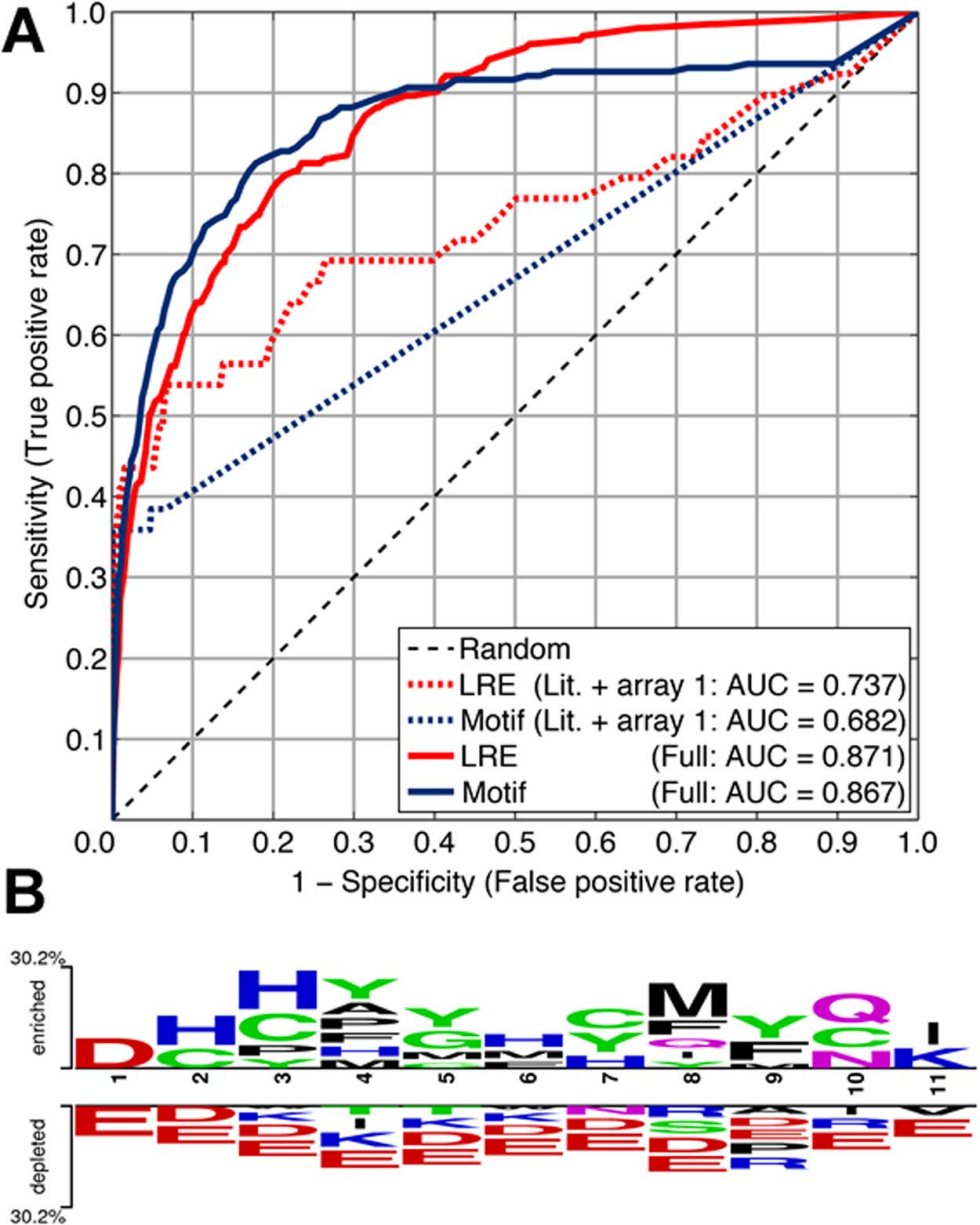
(**A**) ROC curves for the motif-based and logistic regression ensemble (LRE) predictors described in this study. The dashed lines represent the models’ performance when trained on the literature-derived peptides and peptides from the first array. The solid lines represent the models’ performance when trained on the final combined data set. (**B**) Two-sample logo of all the arginylated peptides in the final combined set against the (restricted) unlabeled peptides. Two Sample Logo was run with default parameters and color settings (no Bonferroni correction was applied).

A combination of all three data sources resulted in a 10-fold increase in the data set size over the original literature set. Furthermore, the selection of peptides with only one D or E residue in the second peptide array experiment introduced more diversity into the data set. Thus, we expected that a model trained on this final combined data set would be more accurate and robust to overfitting. In cross-validation experiments, we found that this was indeed the case. The AUC of the logistic regression ensemble predictor increased to 0.87, which represents a 17.6% increase over the model trained only on the literature and array 2 sets (Fig. 7A). Interestingly, the motif-based predictor also performed comparably with an AUC of 0.87, suggesting that the larger data set enables the identification of a consensus sequence. Two Sample Logo^19^ revealed a motif with a 30% enrichment in the positive set (Fig. 7B). However, this enrichment was insufficient to explain the performance increase observed, and we hypothesized that the motif-based predictor was overfitting the data obtained from the peptide array experiments. To test this hypothesis, we devised an experiment in which both the logistic regression ensemble and motif-based predictor were trained only on the peptides obtained from the array data sets and tested on the original literature-derived peptides. The AUCs of the two predictors were 0.79 and 0.65, respectively, suggesting that the motif-based predictor generalizes poorly. Taken together, our previous conclusion that arginylation is more likely to be driven by a combination of both physicochemical properties and consensus sequence near the site still holds.

We undertook an analysis of the features in our predictor that were most associated and predictive of arginylation. For each feature, we calculated the Kendall’s rank correlation coefficient and assessed the significance of the resulting association. Overall, the correlation coefficients ranged between −0.120 and 0.157, suggesting modest associations of the individual features with arginylation. However, 32 features were found to be statistically significant even after correcting for multiple-hypothesis testing (Table S3). Unsurprisingly, we observed significant correlations for position-specific occurrences of certain residues (as in Fig. 7B). Specifically, histidine and cysteine residues were preferred in the +1 and +2 positions, with a general preference for tyrosines at positions +3 and +4 (Table S3). In the +7 position, the presence of a methionine residue is highly associated with arginylation and is, in fact the third most correlated feature. Apart from positional motif-like preferences, our analyses also revealed that the frequencies of H, C and Y near the D or E (less so for tyrosine) and along the entire peptide are highly predictive of arginylation. In addition, the net charge of the peptide was correlated with arginylation but the total charge was anti-correlated, i.e. arginylated peptides contained very few charged residues such as K, R, D and E but when they did, they were mostly positively charged. This is in general agreement with the fact that the frequency of E was most anti-correlated with arginylation. However, it is likely that this observation arose out of the single-D-or-E constraint that we enforced upon the array 3 set. Taken together with the consensus sequence, our results suggest that N-terminal arginylation generally occurs on mildly positive peptides and/or those that are rich in polar residues.

### Comparison of predictor performance to baselines generated through simulation

An interesting observation in Table S2 is that the proportions of peptides arginylated in the assay were similar across all groups, irrespective of whether they were scored low, medium or high. This could be explained in one of two ways: either the original model is so biased toward the literature and the array 2 data set that it predicts incorrectly, or the assay is insufficiently selective and all peptides are equally likely to be arginylated, irrespective of their sequence. To rule out the latter, we carried out a simulation experiment to evaluate the performance of our predictor had the training data set been collected in a manner different to that of the original protocol. We simulated the arginylation assay on array 2 in silico by randomly assigning arginylation outcomes to the peptides in the array. The mean cross-validation AUC over 1,000 simulation runs was found to be 0.82 with a standard deviation of 0.01, which is quite high but much less than that of the actual predictor.

A related concern was that our overall protocol involved the repeated use of computational models to guide the selection of peptides for assays. This may have the unintended consequence of the generation of a biased training set, which in turn, could lead to a biased model and a spurious improvement in performance, as more training data gets added. To address this, we undertook two more simulation experiments. First, we redesigned array 2 to contain a random set of 222 peptides, instead of those with low, medium and high scores as in the original protocol. This has the effect of decoupling the predictive model from the selection of peptides that would eventually constitute a large proportion of the training data. In this simulation, the mean cross-validation AUC over 1,000 simulation runs was 0.75 with a standard deviation of 0.01, which is even lower than the previous simulation experiment. Second, we redesigned the initial training set (literature plus array 2) to contain peptides randomly selected from the human and mouse proteomes, trained a model and used it to design array 3 (based on the low-, medium- and high-score criteria as before). Experimental outcomes were again randomly assigned and a final model was trained and evaluated. If the mean AUC of these simulation runs was equal to or greater than that of the actual predictor, it would suggest that our study protocol was simply selecting for the biases in the initial training set and amplifying them, and that the apparent improvement in model performance was simply a consequence of this amplification. Over 15 simulation runs, we found the mean cross-validation AUC to be 0.76 with a standard deviation of 0.02, which is much lower than the actual predictor’s AUC value.

### Estimating the scope and evolutionary conservation of the N-terminal arginylome

We ran our final predictor on the human and mouse proteomes to estimate the fractions of D and E residues that may be arginylated. However, the accurate estimation of such fractions is not straightforward because our predictor construction protocol does not ensure that scores output by the predictor are readily interpretable as probabilities. Although an artificial score threshold could be applied, it is always associated with a false positive rate and any estimates derived from it would be subjective. Therefore, we used a protocol developed previously^20^ that relies on the AlphaMax algorithm to estimate proteome-wide fractions^21^. Since the predictor was not trained on a bona fide negative set (peptides that cannot be arginylated), we had to first estimate the fraction of unlabeled peptides that may contain arginylation sites. Furthermore, experimental noise and uncertainties may have led to incorrect identification of arginylation sites (e.g., the assumption that arginylation always occurred on D or E at position one), and the fraction of incorrectly labeled arginylation sites needed to be estimated. Again, for practical purposes we retrained the predictor using the true unlabeled set instead of the restricted unlabeled set and recorded scores in a 10-fold cross-validation experiment (out-of-bag scores yielded similar results).

Using the AlphaMax algorithm, we estimated that 6% of 10,000 D and E residues randomly sampled from the human and mouse proteomes that constituted the true unlabeled set may have been undiscovered arginylation sites, and that 8.5% of our positive set may be incorrectly labeled arginylation sites. Next, this predictor was run on the two proteomes to obtain a score distribution for each species, which was, in turn, transformed into a posterior probability distribution by using the above fractions in the expression detailed in^21^. Finally, the mean of this posterior distribution was calculated to derive the empirical prior probability that a D or E residue in the proteome is arginylated; i.e., the fraction of arginylated D and E residues. We found that, in both the human and mouse proteomes, the fraction of potential arginylated D and E residues was up to 3%. Given that the numbers of D and E residues in the human and mouse proteomes are 1,340,938 and 1,125,895 respectively, this amounts to substantially large numbers of potential arginylation sites at the proteome level (nearly 40,000 in human and over 33,000 in mouse). We note that the predictor was trained to recognize local features around the arginylation site and does not account for whole-protein features. Moreover, it assumes that every D or E residue input to it occurs at the first position, thus being N-terminally exposed. It is unclear to what extent these assumptions affect our estimates. To derive more conservative estimates, we considered only those D and E residues that occurred at the first or second position of a protein because these are more likely to be N-terminally exposed *in vivo*. This yielded a subset of the two reference proteomes with 3,146 residues in human and 2,680 in mouse. The fractions estimated on these sets were 2.6% and 2.5% for human and mouse, respectively (about 70–80 residues/proteins). Although this is a much smaller number, it is extremely conservative, given that D and E residues can be N-terminally exposed through signal peptide and proteolytic cleavage and can also undergo side-chain arginylation by ATE1^10^. The proteins identified in this analysis of mouse and human genomes are listed in Tables S4–S7.

Independently, we also analyzed the alignment of protein coding sequences, compiled from 29 mammalian genomes^22^ to identify all proteins with evolutionarily conserved N-terminal MD, ME, and MC sequences in mammals. This analysis revealed 1,485 proteins, listed in the Tables S8–S10. Of these, the number of proteins with the conserved C in second position was by far the lowest–62 proteins (Table S8), compared to 510 proteins with conserved D (Table S9), and 913 proteins with conserved E (Table S10).

To narrow down this analysis to a shorter list of likely arginylation targets with likely conserved regulation across mammals, we extended our analysis of the mammalian alignments to encompass the larger 12-codon motif for all proteins bearing N-terminal MD or ME, matching the algorithmic predictions and the consensus peptide length. From 1480 MD-containing and 2441 ME-containing human proteins at their N-termini, we used the breadth and strength of conservation to select 1024 candidates with an N-terminal MD (Table S11) and 1616 candidates with an N-terminal ME (Table S12). These candidate hits were further trimmed by only selecting those that showed above-threshold scores using the arginylation consensus motif, yielding a list of 24 proteins that are highly likely regulated by N-terminal arginylation in mammals (Table S13). Testing the N-terminal sequences of these targets by our algorithm revealed an overall high arginylation score for each protein, ranging from ~0.78 to ~0.98 (Table S13). These scores further support our hypothesis that these protein targets may be highly regulated by N-terminal arginylation *in vivo*.

## Discussion

In this work, we systematically dissected the N-terminal target site specificity of the four mammalian ATE1 isoforms using *in vitro* assays with the oriented immobilized peptide arrays, and demonstrated that these four ATE1 isoforms have different, even if partially overlapping, preference for their target sites. We show that the four ATE1 isoforms show different preferences for the N-terminal and midchain target sites and are potentially able to arginylate “non-canonical” target sites, including unoxidized C and potentially other residues. Furthermore, we combined targeted peptide array designs and *in vitro* arginylation assays to develop an algorithm for prediction of proteins arginylated *in vivo*. Although, due to the limitations of the peptide array, this algorithm has been based on prediction of the “canonical” N-terminal arginylation at D or E, it is likely that similar prediction strategies can be applied to “non-canonical” target sites, including midchain D/E arginylation and other residues at the N-termini.

A recent study using the same method, but different peptide designs, reported that the four ATE1 isoforms appear to have similar target site specificity and preference for residues in the adjoining positions to the N-terminal target sites^13^. In the current study, our different peptide design enabled us to test the broader target site specificity of ATE1 isoforms and find prominent differences between them, as well as to perform detailed analysis of the arginylation consensus motif. Notably, this same group previously reported that all four ATE1 isoforms have similar activity toward N-terminal D/E containing substrates^23^, while our studies consistently find that purified ATE1-3 and ATE1-4 are weaker in *in vitro* assays than ATE1-1 and ATE1-2, and are potentially more active in mid-chain arginylation^10,14^. Understanding the underlying reasons for these discrepancies would likely uncover important mechanisms of regulation of ATE1 activity.

At present, nothing is known about the mechanisms of intracellular targeting of ATE1 to specific protein substrates. It has been estimated that hundreds, possibly thousands of proteins, can be arginylated *in vivo*^9^, but the identification and functional analysis of these proteins remained elusive. Our results suggest that the identity of the amino acid residues around the arginylation target sites is likely important for ATE1 targeting and can potentially be used in reliable predictions of proteins arginylated *in vivo*. Using these predictions to perform targeted identification of ATE1 protein and peptide substrates constitutes an exciting direction of further studies.

While the peptide arrays provide a powerful tool for testing a multitude of ATE1 peptide substrates in a standardized assay, their use does not replace the necessity for the more physiological tools. For example, our prior study suggests that, when possible, arginylation of a peptide *in vitro* would result in a linkage of R to the peptide’s N-terminally exposed alpha amino group^10^. At the same time, this prior study also suggests that ATE1, when presented with protein rather than peptide substrates, preferentially links R linkage on the side chains of D and E, rather than the N-terminal alpha amino group. Thus, the peptide array format, where the orientation and the conformation of the target sites are both severely constrained, can yield only limited information about *in vivo* arginylation. Based on our data shown here, we believe that many of the “non-canonical” arginylated residues previously seen in our *in vivo* screens are likely targeted by ATE1-1 in the presence of these *in vivo* cofactors. This possibility requires further studies.

Much of the controversy in the recent literature revolved around the arginylation of C, and the proposed requirement for oxidation to enable this arginylation. A previous study proposed that C oxidation and arginylation, followed by degradation of the target substrate, serves as a mechanism for oxygen sensing^7^. While our data overall support this mechanism and demonstrate that C trioxidation greatly enhances arginylation, they also suggest that this may be a dynamic dosage effect rather than an all-or-nothing response.

While arginylation of “non-canonical” target sites does occur *in vivo*, and is apparently ATE1-mediated, it is clear that such arginylation is far less preferable to the arginylation of N-terminally exposed acidic residues in proteins and peptides. Such “arginylation-favorable” N-termini can be generated by multiple mechanisms in cells, including the action of D/E specific aminopeptidases, proteases that cleave off signal peptides, as well as proteases that cut N-terminally to D and E – e.g., a number of caspases. With all these mechanisms, almost every D or E (or C) residue can in principle be exposed during protein N-terminal processing, functional cleavage, or degradation. Thus, our algorithm provides a useful and timely tool for the prediction and global analysis of protein arginylation.

Testing our algorithm against some of the previously used test substrates for arginylation (including X-beta-Gal and X-nsp4 utilized in many of the N-end rule degradation studies) shows surprisingly low scores for these N-terminal substrates (0.2327 for D-beta-Gal and 0.5992 for D-nsp4). These scores are substantially lower than those for the peptides used in this study, or the predicted 24 highly regulated mammalian targets listed in Table S13. It is possible that, while these proteins may be arginylated in the *in vitro* overexpression assays, they may not be optimal for the arginylation reaction. Even lower scores were obtained for the N-terminal sequences of RGS4, RGS5, and RGS15, previously shown to be arginylated *in vivo* after removal of N-terminal M and trioxidation of N-terminal C^7^. These scores, obtained by substitution of C with D for computational purposes, equal 0.0859, 0.1561, and 0.2020 for RGS4, 5, and 16, respectively – far lower than any of our positive hits. It is possible that in the case of N-terminal C the arginylation recognition consensus may be entirely different, not applicable to our algorithm. It is also possible that the RGS proteins are lower efficiency targets for arginylation.

A body of work over the years proposed arginylation as a branch of the N-end rule pathway of protein degradation, in which addition of R to the N-terminus of proteins and peptides facilitates its ubiquitination and removal by the proteasome^24–26^. While this, unquestionably, happens for some proteins, a growing body of evidence suggests that many proteins *in vivo* are not substantially destabilized by arginylation^27–30^. Moreover, our recent work showed that some pairs of homologous protein isoforms can be differentially arginylated because of changes in their coding sequence^31,32^, resulting in their differential targeting for either degradation (in the case of the incorrectly arginylated isoform) or survival and functional regulation (for the correctly arginylated one). It appears likely that the choice of this fate must depend, at least in part, on the sequence context around the target site, and potentially be mediated by different arginylated residues. Unraveling the complexity of arginylation-dependent protein regulation is the essential next step in resolving the current controversies and understanding the place of this mechanism in global posttranslational control of protein functions.

## Methods

### Peptide arrays

Peptide arrays were synthesized by the Biopolymers Laboratory, Koch Institute for Integrative Cancer Research, Massachusetts Institute of Technology. In brief, the 10 × 15 cm membranes were synthesized using SPOT synthesis technology^33^ on an INTAVIS Bioanalytical Instruments AG (Köln, Germany) model Spotter cellulose peptide array synthesizer. The Intavis Spotter consists of a computer-controlled diluter and XYZ liquid handling robot which allows the deposition on amino-PEG derivatized cellulose membranes of individual activated amino acids resulting in peptide formation using standard FMOC HOBT/DCI chemistry. Instrument method was modified slightly to increase the number of coupling cycles and FMOC deprotection steps per coupling cycle.

After synthesis the membrane was treated with trifluoroacetic acid/ triisopropylsilane/H_2_O 92.5/5/2.5 cocktail for two hrs. to remove amino acid side chain protection groups. After removal of cocktail the membrane was subjected to three x 2 min. washes of dichloromethane, three x 2 min. washes of ethanol, three x 2 min. washes of H_2_O and two x 2 min. washes of ethanol. The membrane was then air dried and gridded with pencil.

For quality control, a few blank spots on each array were programmed for synthesis with a C-terminal Rink Amide Linker residue. This linker allows the spot to be cleaved off the membrane in our TFA cocktail along with all of the protecting groups and leaves a C-terminal amide on the test spot peptides. Each of these spots was then cleaved for 2 hours in 100 ul of 92.5%Trifluoroacetic acid, 5% Triisopropylsilane and 2.5% water for 2 hours. 20% of the cocktail was dried down, reconstituted with 1 microliter of CHCA maldi matrix solution, and spotted on the plate for MALDI mass spectrometry analysis.

### *In vitro* arginylation assays

*In vitro* arginylation assays were performed as previously described^10,14^. For the assays in solution, the peptides after the arginylation reaction were purified on a reverse phase column and analyzed by MALDI-TOF or LC MS/MS mass spectrometry (see below) of by scintillation counting. For the assays on the membrane, the whole membrane with immobilized peptides was pre –blocked with 0.5% Acetylated BSA and 1 mg/ml total tRNA in 1 x arginylation reaction buffer at 37 °C for 1 hour, then incubated in the arginylation reaction mixture at 37 °C for another 1 hour, followed by treatment with 0.5 mg/ml ribonuclease A at 37 °C for 30 minutes and triple washes with PBS buffer. The membrane was air dried on 3 M filter paper and exposure to the X-ray film. Due to the strength of the signal, 12–24 hour film exposures are shown in Figs 2–6.

#### Mass spectrometry

Peptide samples were desalted by stage tipping and dried down in a vacuum concentrator. After resuspension in 0.1% TFA in water, peptides were loaded onto a 75-µm I.D × 17-cm in-house-packed column (ReproSil-Pur C18-AQ; 3-µm particle size) using an Easy-nLC-1000 (Thermo Scientific). Peptides were eluted over a 75-min gradient with 3%–32% solvent B (A- 0.1% formic acid, B- 80% acetonitrile, 0.1% formic acid) with a flow rate of 500 nL/min. The nLC was coupled to an Orbitrap Fusion (Thermo Scientific) operating in data-dependent mode (DDA). The spray voltage was set at 2.3 kV, and the capillary temperature was set at 275 °C. Full MS scans were performed within the range of 350–1600 m/z at a resolution of 120,000 (at 200 m/z). Data-dependent MS/MS was performed in the ion trap using top-speed mode set to 2 sec; signals with charge state 1–4+ with an intensity >10,000 counts were automatically selected for MS/MS fragmentation performed using high-energy collision dissociation (HCD) with normalized collision energy of 27. MS raw files were analyzed using Proteome Discoverer 2.2. Parameters for MS/MS database searching included the following: precursor mass tolerance: 10 ppm, product mass tolerance: 0.2 Da, enzyme: unspecific, static modifications: ^13^C(6)^15^N(4) (R), variable modifications: carbamidomethyl (C) and oxidation (C, M), dioxidation (C), trioxidation (C). Each peptide was individually searched using the SEQUEST algorithm against a database containing the native peptide sequence as well as that containing an N-terminal arginine residue. Peptide-spectrum matches with a delta Cn value ≥ 0.05 were used for validation. Only species corresponding to the full-length peptide sequence (plus or minus an N-terminal arginine) were considered.

Algorithm development and arginylation predictor construction are described in detail in Dataset 2.

#### Conservation analysis of N-terminal arginylatable sites

In order to narrow the scope of possible targets, N-terminal arginylatable sites were analyzed for their conservation in the mammalian lineage. The genomic regions for all CCDS transcripts (CCDS Homo Sapiens release 9) were analyzed using the 29 mammal alignment^22^. The alignments for the first 12 codons, to match the consensus peptide length, were used to compute PhyloCSF scores^34^ as a measure of conservation. The raw data can be found in Tables S11 and 12. Transcripts with sufficiently good alignments (branch length scores greater than 0.4), complete conservation of the arginylatable residue (D or E), and positive PhyloCSF scores were matched with scores computed from the consensus motif. This list of candidates was trimmed to include only one transcript entry per gene and can be found in Table S13. Entries with the highest PhyloCSF and consensus scores are the most likely to be arginylated.

## Electronic supplementary material

Supplement

Table S4

Table S5

Table S6

Table S7

Table S8

Table S9

Table S10

Table S11

Table S12

Table S13
