# Supplementary material for: Target site specificity and in vivo complexity of the mammalian arginylome
Source: Sci Rep. 2018 Nov 1;8:16177. doi: 10.1038/s41598-018-34639-6 (PMC6212499; doi:10.1038/s41598-018-34639-6)
Supplement: Supplementary file 1 — Supplement [file 41598_2018_34639_MOESM1_ESM.pdf]

Target site specificity and *in vivo* complexity of the mammalian arginylome

Junling Wang, Vikas Rao Pejaver, Geoffrey P. Dann, Max Y. Wolf, Manolis Kellis, Yun Huang, Benjamin A. Garcia, Predrag Radivojac, and Anna Kashina

Supplemental Online Information.

Wang_et_al_FigS1

Side chain arginylation	N-terminal site specificity	Natural peptides
S**D**IAALVHSGMC	**G**IAALVHSSGMC	**L**YANNVLSGGTT
SI**D**AALVHSGMC	**A**IAALVHSSGMC	L**E**VLNFFNNQI**E**
SIA**D**ALVHSGMC	**L**IAALVHSSGMC	SYVG**DE**AQSKMC
SIAA**D**LVHSGMC	**M**IAALVHSSGMC	M**D**PLN**D**NIATLL
SIAAL**D**VHSGMC	**F**IAALVHSSGMC	KPVY**DE**LFYTLS
SIAALV**D**HSGMC	**W**IAALVHSSGMC	G**DD**GA**E**YVV**E**ST
SIAALVH**D**SGMC	**K**IAALVHSSGMC	**N**SALQCLSNTAP
SIAALVHS**D**GMC	**Q**IAALVHSSGMC	LQ**EE**IAFLKGMC
SIAALVHSG**D**MC	**E**IAALVHSSGMC	FYN**E**LRVAP**EE**H
SIAALVHSGM**D**C	**S**IAALVHSSGMC	**E**HC**D**CLQGFQLT
SIAALVHSGMC**D**	**P**IAALVHSSGMC	HSQAV**EE**LA**D**QL
S**E**IAALVHSGMC	**V**IAALVHSSGMC	MC**D**RKAVIKNA**D**
SI**E**AALVHSGMC	**I**IAALVHSSGMC	**D**IARQVG**ED**CRT
SIA**E**ALVHSGMC	**C**IAALVHSSGMC	CQSHRLTV**ED**PV
SIAA**E**LVHSGMC	**Y**IAALVHSSGMC	S**E**AGA**E**YVV**E**ST
SIAAL**E**VHSGMC	**H**IAALVHSSGMC	
SIAALV**E**HSGMC	**R**IAALVHSSGMC	
SIAALVH**E**SGMC	**N**IAALVHSSGMC	
SIAALVHS**E**GMC	**D**IAALVHSSGMC	
SIAALVHSG**E**MC	**T**IAALVHSSGMC	
SIAALVHSGM**E**C		
SIAALVHSGMC**E**		

Figure S1. Design of the initial peptide array shown in Figs 1-3. Bold underlined residue indicates the most likely arginylation target site. Peptides are shown from N- to C-terminus, with the N-terminus exposed on the array.

Wang_et_al_FigS2

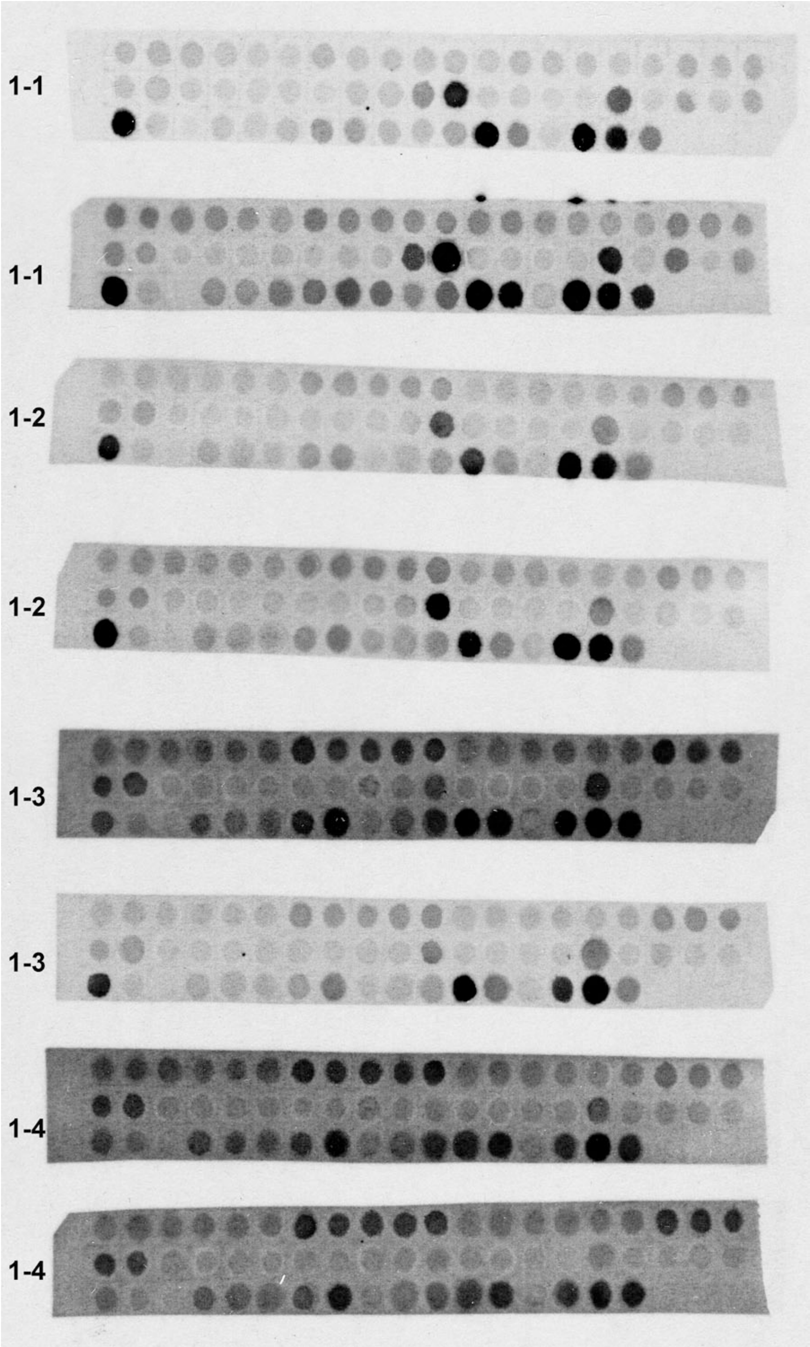

Figure S2. Raw image of the initial peptide array 1 shown in Figs 1-3. In the main text figure, gray levels were adjusted to the same background to enable direct comparison of signal intensities on the array.

Wang_et_al_FigS3

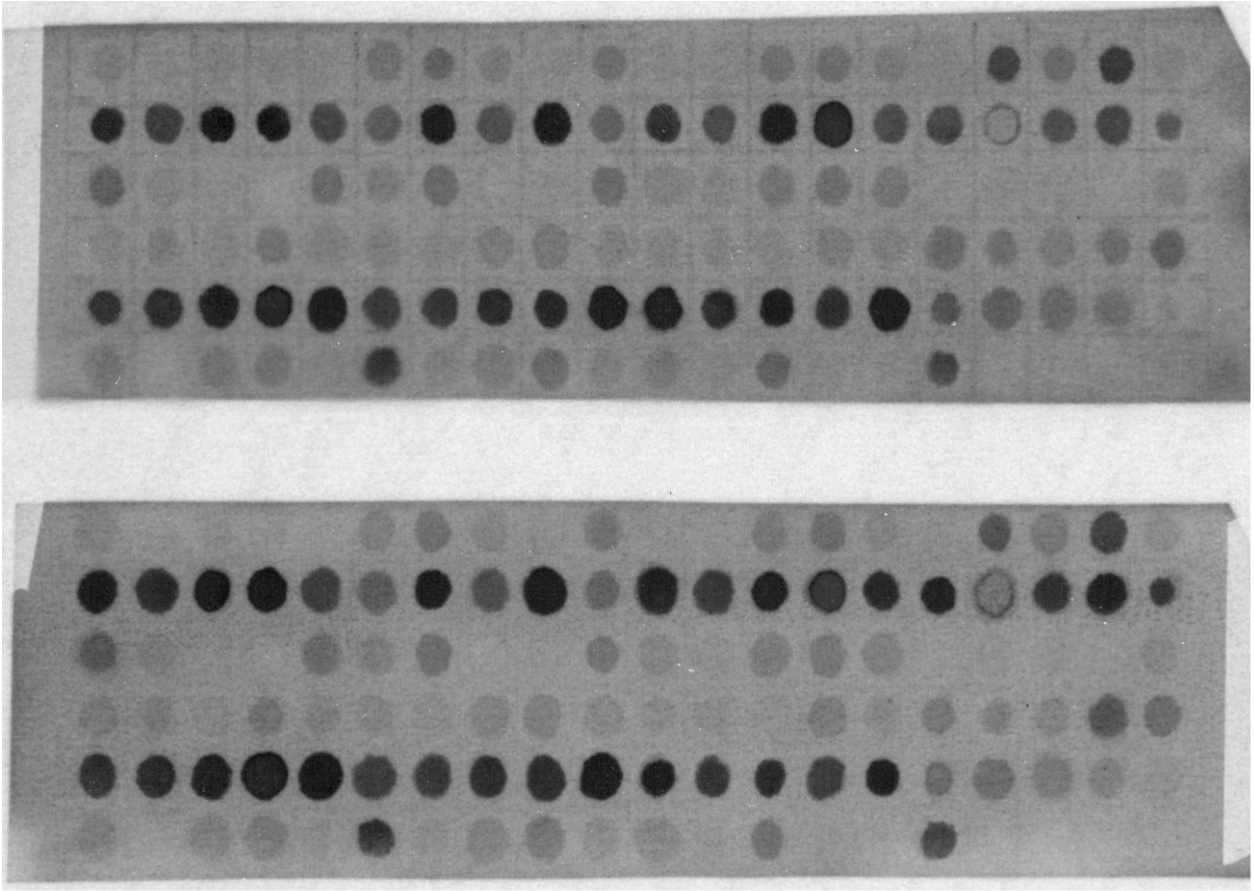

Figure S3. Raw image of the array 2 shown in Fig. 5. In the main text figure, gray levels were adjusted to the same background to enable direct comparison of signal intensities on the array.

Wang_et_al_FigS4.

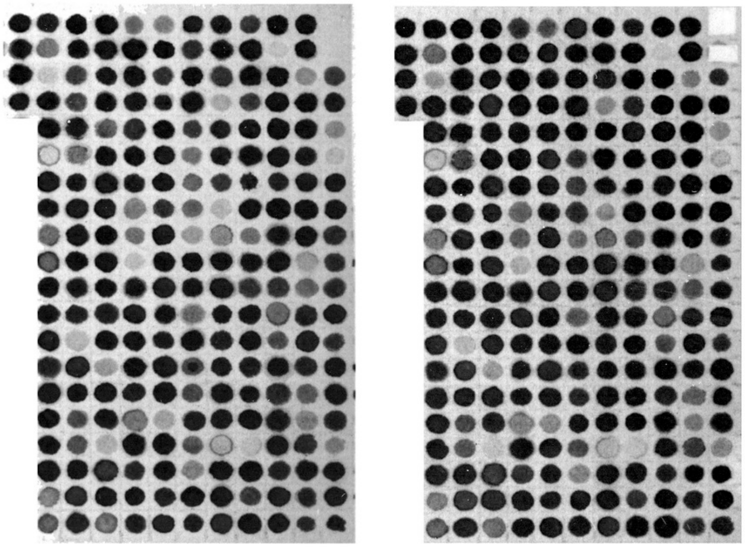

DVGGAQQCGMR	DHLMNVLVVWI	EFGFLSKLVYR	ETTSFYVLNVT	DRFPPMMHHHS	EQHFQHGGYQY
ELNHYNVATGK	ELTTQLTIIMI	ETCCAAGQQWA	DGHYQTVQCQT	EHSFNNFQMKI	DKHYCHCQAGY
DVHAFQQFNWK	DIPQFMRQMAL	DGFHVYQKGCF	EAAFMYVFYFL	DTHYYRFKLTN	EVHHCYYQICK
ENPMVQQFIQR	EQCTRGSLFMC	EKIASNSKIVV	DCKTIRIIYSI	DLMYGGIYCFL	DCGHGMVMLMA
	EVNNCSICYHW	DPRCFLHMSVQ	DHIFKYTNFGI	EFCFRFCINHL	DRKPTTGHFQR
	DAMYVIMWVTY	ENMVRMAPPYP	DVHCLSHPYFK	DPPYTHTIQKQ	DWCLHHICTNY
	DIFPFKKLVNF	DVNGLPYGLCA	ERHHSHKRRKQ	EFPGQAVASVI	DACFGMSAIQM
	DPIMGFHQMFL	EQRHIVRILFI	EPPFVHYLNTF	EKPYAVAMQYL	EHAMANKQVCK
	EHVVRSRIQTS	EHSNRPINVIK	DRFNYVVMQLQ	ETHMPVYCSCA	ELCCYRYHVTA
	DRQYVVRLMAP	EILARPKPWNK	EHLHHLYFRAR	DARYMKGCLNM	EAHPYSTMHNS
	DMMVSIFYSIV	EVRHISHLNFT	DCVSCQPYVVK	ERKMQMPFQKG	DHAYALRMLPK
	DTCFCCLITWL	DRYAVYWNSSN	DQHVRQQFLQG	EQKPFVCGVCK	DHGFPNHQYNS
	DPNYLHRMTTL	ECASINRIYGF	DHHWTNMTVQC	DHCKYRYLFNF	DKMYNHYIIKK
	DIHQGFGNLLQ	DPHSVHSVHGP	DFVMMHCVFMP	ECHCSTCYYHK	DCHPFSLSPNT
	DISVIVIYFVV	EYSGPLLIYLL	DSPPTPTMYKY	DCCPQYSFQLQ	ESSAYPYTYGT
	ESTYGLYRTHL	DFFQYGSYVCT	DRNLVVTQCGN	DHMGGFYLCQK	EGCFNRHCHVP
	EWMMIHRILNH	DRPPYGVFTIN	DNCYCSSVANH	DICPMKYSSCP	DHMVSGANMTI
	ECRKICKQRKG	DARFRMFCKQG	DCGNNRVGVVS	DYCARVCAMSI	DRCQNQCFTKR
	ETIKFSHCAVI	DRAFHFTAMPK	DIHVLAMMYSG	DPFAFFGMQAP	DGPNPPYMVLL
	EQYRRSQIQAV	EHMMPLCMVIS	DTPFRLYGLTM	DCHYRYYLYNL	DRYRMTKMHSH

EKPNGLLYFQH	EVHQNFSAWCS	EVFNGYFVHFF	DFLAMFSPKCG	ENHHLRHGGRM	EGHSYYYISKP
EHYFMPFGKGW	DFQPHVIVHCA	DTCNKQLPKPA	EVGHQGVVFQH	ECHAFLCTKRK	ETMYQHYYFQG
DHKRMFGTYFR	EAFHCISSFHK	ELCQFFQQCLC	DRPAYLWLKQR	DKCPYMAFHNN	DILQNPLMIKI
DGVRRNCTFQR	EVIVRIIGHFF	DWIILILTFYT	DLYTCRPAYTP	ECCFTYTTYKI	DRHLYIVTGRC
EHFFWSFMLYG	DLRSPNCQGLM	DCYYCVSGLTQ	DLALQRHKHHM	DKCYKCVVKQG	EIHTYMKMFQR
DCHKCRYLCTG	ESTVYSRMIQA	EAHNMGTIHMS	DRFNYVVMQLQ	EHFFYYYYYYT	DLMGPFHTSNR
DLCPCRHNGQW	EMGGHHIVALC	DCPKMMFIHAT	DFCCPKCQYQA	ECFCRSCFVTS	DGYRIGFGQCA
DMAMSNIVGSN	DNLAPITAKAK	DSCPASRATCV	EHLAKKKYHCT	DCAPNNGYFMI	DRHMYHSLYLK
EKAMIAKMNRQ	DVNNSTLMFVG	DRIHIPIFHGR	DKYFYIQMQIC	DKHYGFQLYQS	ETTMLGGYVYS
EACKMVNYMQY	DFVGCAQGMCC	DVGRMFKQFNK	DVVYVAQMIQK	DCHYRYYLYNL	DHCLCKCAQVM
ETTGHGFPHFS	DLALWPVIYSC	DYYARLFGIKS	DLFYQTTQKGT	EACWRFAFYLM	DRCQNQCFTKR
EKAKSHQTWKA	DVGANIFIGNL	DHKSTSHFRTG	DAFMRRKCQQC	ELHPYMFHGGM	DGMNSIARGQK
DPSVYRFCFAW	ERTNTRAKSLG	ETPAMMFPHQL	DQPQVPCIFCL	EIPFIQHIYQK	DNHMQLGMICY
DRQTNHCYIAI	EKMVSISNYPL	DNQIPLLVIGT	DKTVYQSIQPI	DAHAPHHPVVH	DHLHQTGMYQV
DIKGVFRYCNT	DLMIRNIQLKH	DYHSYMLQISK	DHQFYMRFTAS	ELCHVCCMKKM	DKKNMGKQMPQ
EGCNHMQCAKC	DSTCRLVSGNL	DHQPSGPYYGH	EHYVSMYIHGN	EVIPYTPAMQR	DRKAPSRINNP
ELYSFLHHQNR	DPMGQQTCSAH	DKCNPLVNLNY	EQVYNACAKQI	EALCGQTFAKK	EHHLQNNKFRI
DCYPAVQKTTL	ELTMSTLINQI	ECHFRFRHQMA	EGIYNCYIMNP	DKKMSGGQSGY	DHHPIVFWNLV
EIHLIGKCLKF	DFRMHFMNYQR	ETFHGFYKSTY	DTKKPKVMVPP	DGHYAVAAKCY	
DSSNMSLWTQN	EKTFQFLHQFS	DPLHYHHLTYI	EQAAQCYIKYI	EHHSYMGMRGF	

Figure S4. Arginylation of the peptide array 3 generated using the finalized predictor algorithm. Two repeats are shown, with the peptide sequences on the array listed underneath.

Table S1: Summary of the positive data sets used in constructing the N-terminal arginylation predictor

**Data set**	**Number of N-terminal arginylated D/E (number of proteins)**	**Number of N-terminal arginylated D/E after removing identical peptides (number of proteins)[a]**
Literature	20 (14)	20 (14)
Array 1[b]	36 (20)	20 (16)
Array 2	188 (188)	163 (163)
Final (total)	244 (222)	203 (193)

[a] Redundancy removal was performed on the final data set and each row represents the contribution of the data set to the full non-redundant data set

[b] Includes 16 N-terminal arginylated D and E residues were detected in synthetic peptides

Table S2: Predictions of the percentage of proteins arginylated in the human and mouse genome.

Species	Score category	Number of arginylated peptides (Percentage)[a]	Number of peptides not arginylated (Percentage)
Human	Low	31 (83.8)	6 (16.2)
	Medium	35 (94.6)	2 (5.4)
	High	30 (81.1)	7 (18.9)
Mouse	Low	30 (81.1)	7 (18.9)
	Medium	34 (91.9)	3 (8.1)
	High	33 (89.2)	4 (10.8)

[a] The total number of arginylated peptides is 193 and was different from that in Table S1 because of duplication (across data sets and between human and mouse) when selecting peptides.

Table S3: Top correlated and anti-correlated features identified by Kendall's rank correlation analysis. Bonferroni corrected P-values at a significance level of 0.05 are shown here.

Feature (window size)	Correlation co-efficient	P-value
H at position +2	0.157	1.4×10^{-20}
Frequency of H (7)	0.139	4.1×10^{-16}
M at position +7	0.137	2.0×10^{-15}
H at position +1	0.126	5.0×10^{-13}
Frequency of H (11)	0.121	2.7×10^{-12}
C at position +2	0.110	1.1×10^{-9}
Frequency of H (3)	0.102	3.4×10^{-8}
Q at position +9	0.101	6.5×10^{-8}
Y at position +8	0.092	1.8×10^{-6}
C at position +1	0.085	2.7×10^{-5}
Y at position +3	0.084	3.9×10^{-5}
C at position +6	0.083	4.8×10^{-5}
C at position +9	0.082	7.1×10^{-5}
Y at position +4	0.081	9.5×10^{-5}
Y at position +6	0.081	1.0×10^{-4}
I at position +10	0.078	2.6×10^{-4}
F at position +8	0.074	8.3×10^{-4}
Frequency of C (7)	0.073	9.2×10^{-4}

K at position +10	0.066	9.9×10^{-3}
N at position +9	0.066	0.01
Frequency of C (3)	0.065	0.01
Frequency of C (11)	0.065	8.7×10^{-3}
H at position +5	0.063	0.02
Net charge (11)	0.063	2.0×10^{-3}
Frequency of Y (11)	0.061	0.03
G at position +4	0.061	0.04
H at position +6	0.060	0.05
Frequency of E (3)	-0.068	2.9×10^{-3}
Frequency of E (7)	-0.092	1.7×10^{-7}
Total charge (7)	-0.098	3.1×10^{-9}
Frequency of E (11)	-0.109	2.4×10^{-11}
Total charge (11)	-0.120	4.6×10^{-15}

Supplemental Dataset 1

#1	b⁺	b²⁺	Seq.	y⁺	y²⁺	#2
1	167.11666	84.06197	R-Label:13C(6)15N(4)			13
2	282.1436	141.57544	D	1260.57112	630.7892	12
3	395.22766	198.11747	I	1145.54418	573.27573	11
4	466.26478	233.63603	A	1032.46011	516.73369	10
5	537.30189	269.15458	A	961.423	481.21514	9
6	650.38595	325.69662	L	890.38588	445.69658	8
7	749.45437	375.23082	V	777.30182	389.15455	7
8	886.51328	443.76028	H	678.23341	339.62034	6
9	973.54531	487.27629	S	541.17449	271.09089	5
10	1060.57734	530.79231	S	454.14247	227.57487	4
11	1117.5988	559.30304	G	367.11044	184.05886	3
12	1248.63929	624.82328	M	310.08897	155.54813	2
13			C-Carbamidomethyl	179.04849	90.02788	1

DIAALVHSSGMC
(arginylated on N-terminal D)

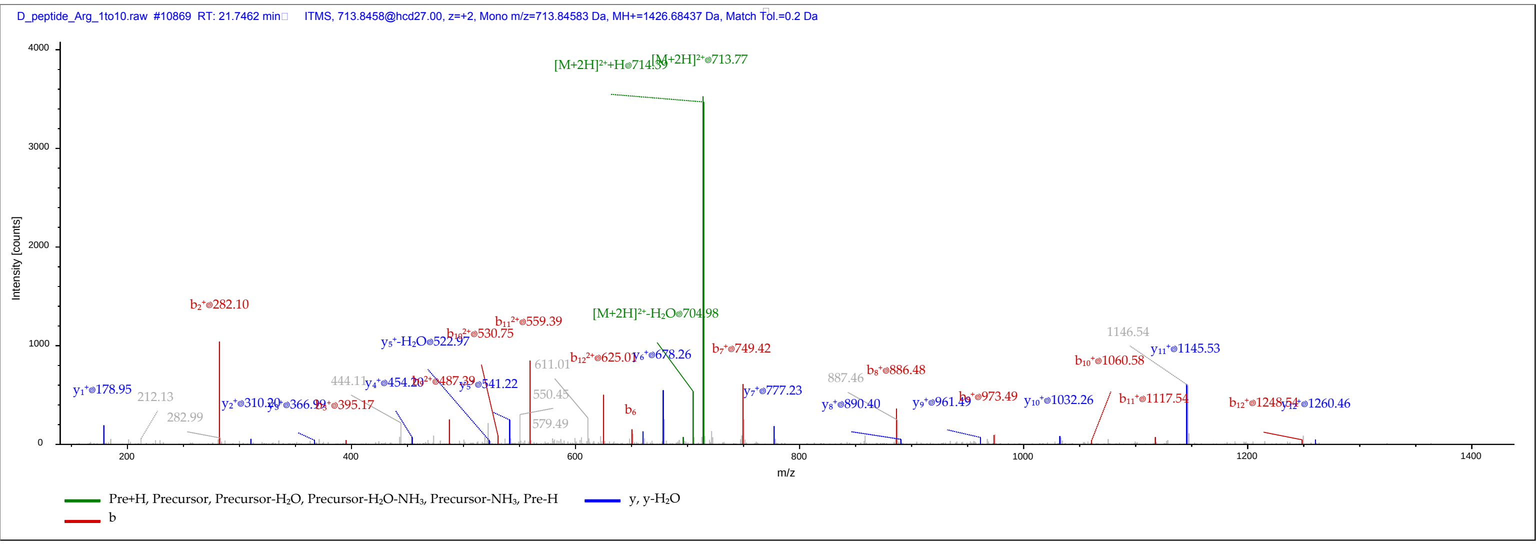

#1	b⁺	b²⁺	Seq.	y⁺	y²⁺	#2
1	167.11666	84.06197	R-Label:13C(6)15N(4)			13
2	318.11059	159.55893	C-Trioxidation	1312.53302	656.77015	12
3	431.19465	216.10096	I	1161.53909	581.27318	11
4	502.23176	251.61952	A	1048.45503	524.73115	10
5	573.26888	287.13808	A	977.41791	489.21259	9
6	686.35294	343.68011	L	906.3808	453.69404	8
7	785.42135	393.21432	V	793.29674	397.15201	7
8	922.48027	461.74377	H	694.22832	347.6178	6
9	1009.51229	505.25979	S	557.16941	279.08834	5
10	1096.54432	548.7758	S	470.13738	235.57233	4
11	1153.56579	577.28653	G	383.10535	192.05631	3
12	1300.60119	650.80423	M-Oxidation	326.08389	163.54558	2
13			C-Carbamidomethyl	179.04849	90.02788	1

CIAALVHSSGMC
(arginylated on N-terminal trioxidated C)

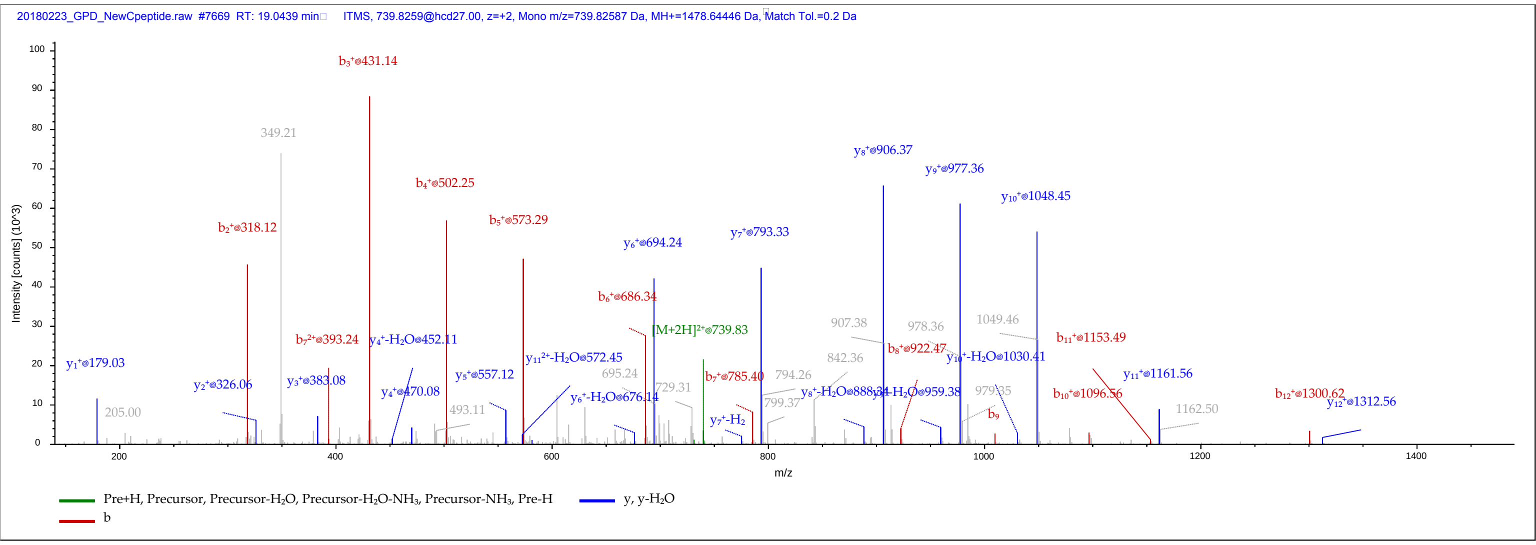

#1	b⁺	b²⁺	Seq.	y⁺	y²⁺	#2
1	167.11666	84.06197	R-Label:13C(6)15N(4)			13
2	327.14731	164.07729	C-Carbamidomethyl	1305.57482	653.29105	12
3	440.23137	220.61932	I	1145.54418	573.27573	11
4	511.26848	256.13788	A	1032.46011	516.73369	10
5	582.3056	291.65644	A	961.423	481.21514	9
6	695.38966	348.19847	L	890.38588	445.69658	8
7	794.45807	397.73268	V	777.30182	389.15455	7
8	931.51699	466.26213	H	678.23341	339.62034	6
9	1018.54901	509.77815	S	541.17449	271.09089	5
10	1105.58104	553.29416	S	454.14247	227.57487	4
11	1162.60251	581.80489	G	367.11044	184.05886	3
12	1293.64299	647.32513	M	310.08897	155.54813	2
13			C-Carbamidomethyl	179.04849	90.02788	1

CIAALVHSSGMC
(arginylated on N-terminal unmodified C)

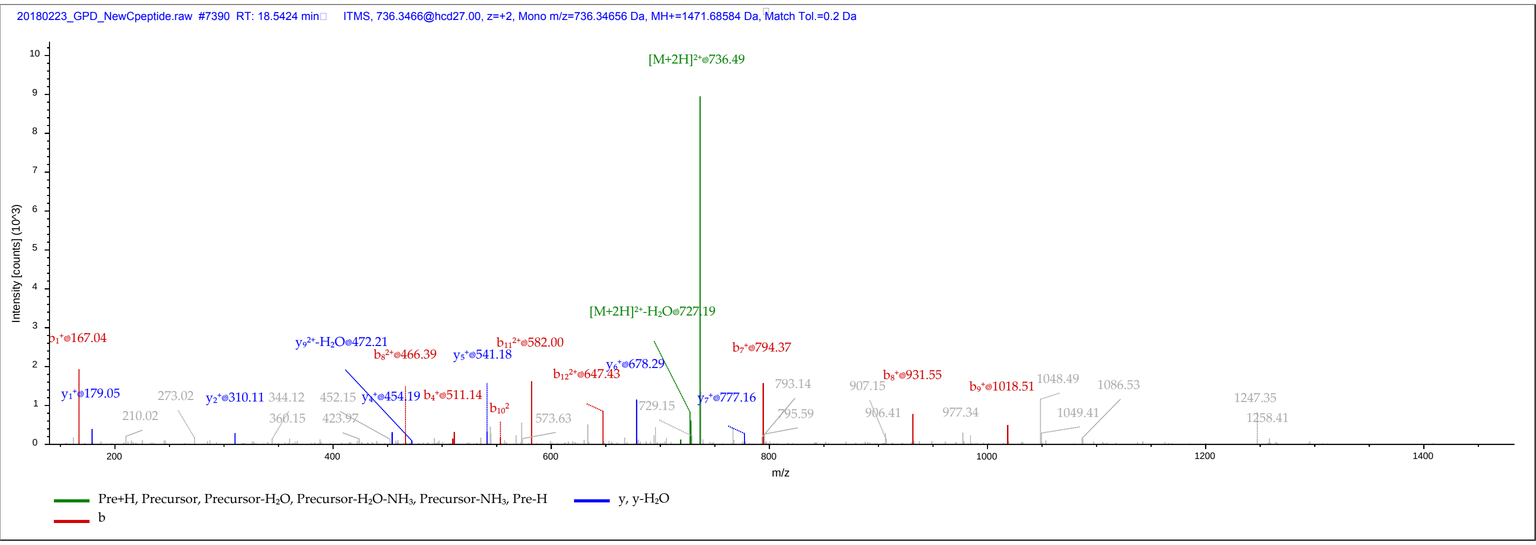

#1	b⁺	b²⁺	Seq.	y⁺	y²⁺	#2
1	152.00121	76.50424	C-Trioxidation			12
2	265.08527	133.04627	I	1161.53909	581.27318	11
3	336.12238	168.56483	A	1048.45503	524.73115	10
4	407.1595	204.08339	A	977.41791	489.21259	9
5	520.24356	260.62542	L	906.3808	453.69404	8
6	619.31197	310.15963	V	793.29674	397.15201	7
7	756.37089	378.68908	H	694.22832	347.6178	6
8	843.40291	422.2051	S	557.16941	279.08834	5
9	930.43494	465.72111	S	470.13738	235.57233	4
10	987.45641	494.23184	G	383.10535	192.05631	3
11	1134.49181	567.74954	M-Oxidation	326.08389	163.54558	2
12			C-Carbamidomethyl	179.04849	90.02788	1

CIAALVHSSGMC
control reaction
(trioxidation)

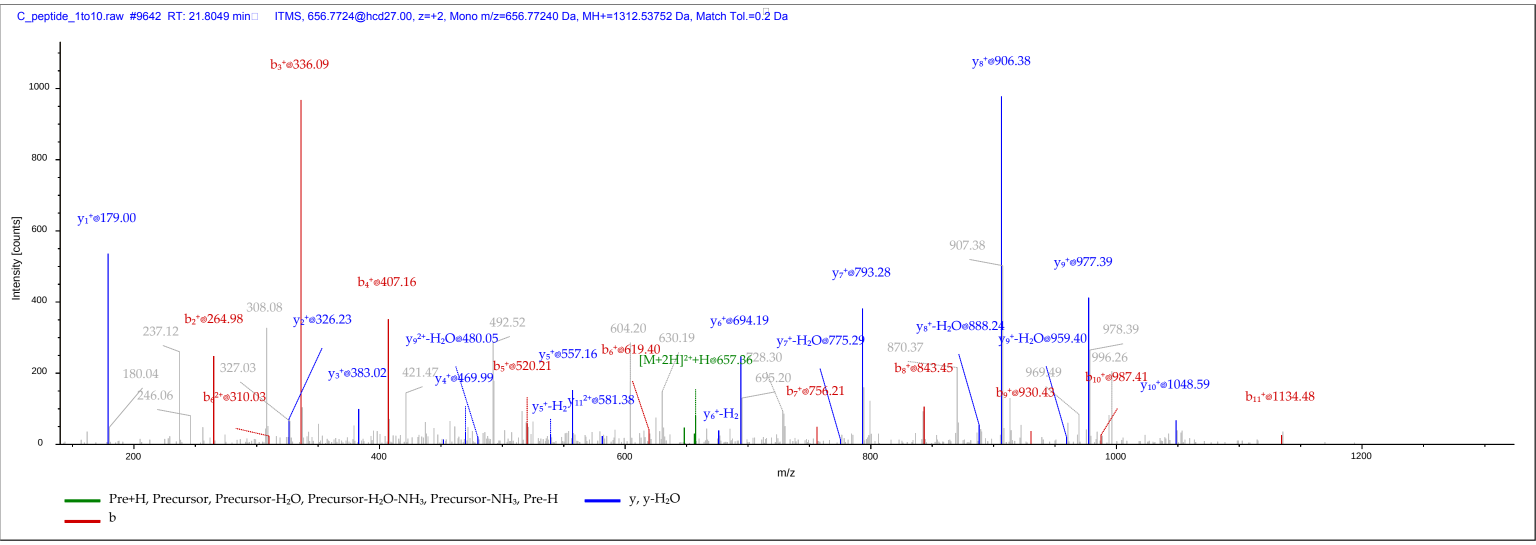

#1	b⁺	b²⁺	Seq.	y⁺	y²⁺	#2
1	161.03793	81.0226	C-Carbamidomethyl			12
2	274.12199	137.56463	I	1145.54418	573.27573	11
3	345.1591	173.08319	A	1032.46011	516.73369	10
4	416.19622	208.60175	A	961.423	481.21514	9
5	529.28028	265.14378	L	890.38588	445.69658	8
6	628.34869	314.67799	V	777.30182	389.15455	7
7	765.40761	383.20744	H	678.23341	339.62034	6
8	852.43963	426.72346	S	541.17449	271.09089	5
9	939.47166	470.23947	S	454.14247	227.57487	4
10	996.49313	498.7502	G	367.11044	184.05886	3
11	1127.53361	564.27044	M	310.08897	155.54813	2
12			C-Carbamidomethyl	179.04849	90.02788	1

CIAALVHSSGMC
control reaction
(no oxidation)

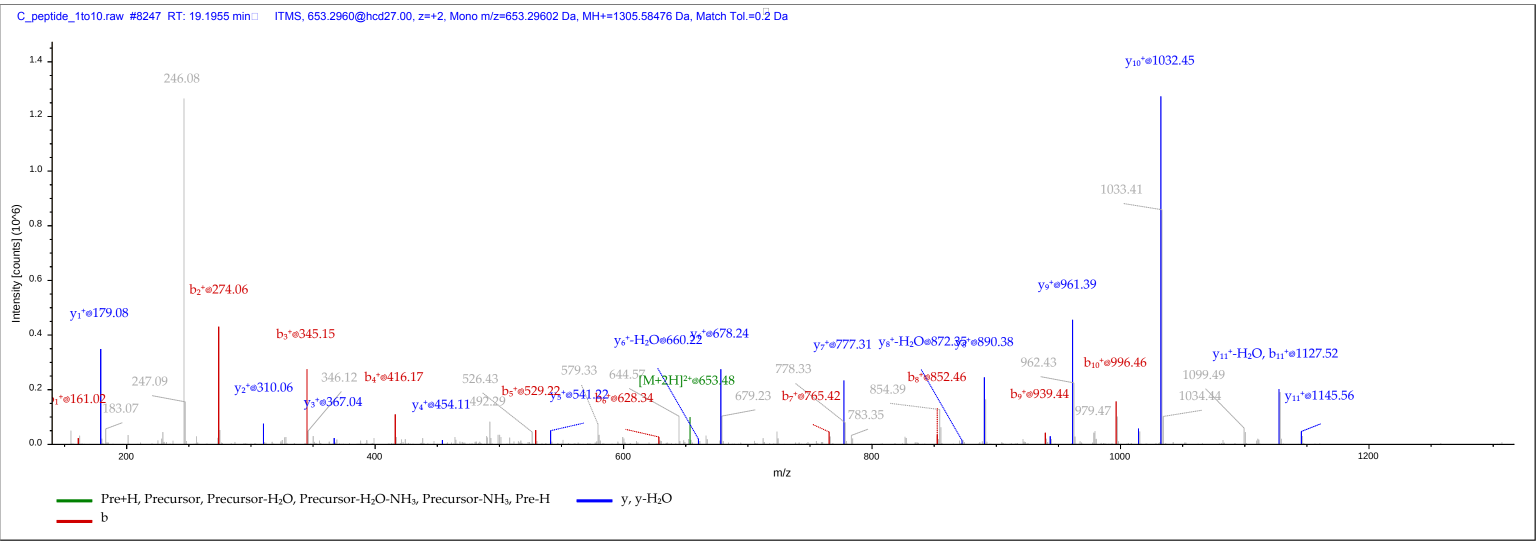

SUPPLEMENTAL DATASET 2

Algorithm development and arginylation predictor construction.

I. Arginylation prediction data sets

Reference proteomes. For all analyses and predictor development, the human and mouse reference proteomes were downloaded from UniProt (release 2016_03). These include only those protein sequences that correspond to the canonical isoform for a gene and have undergone manual annotation and review by UniProt curators. In total, there were 20,193 and 16,768 protein sequences in the human and mouse proteomes, respectively. For the generation of candidates for peptide array experiments, a non-redundant data set of sequences from human and mouse was created by running CD-HIT on all 36,961 sequences combined (Li and Godzik, 2006). This is referred to as the *non-redundant reference proteome* set and contains 14,056 sequences such that no two sequences shared more than 40% identity.

Training data. Experimentally verified N-terminal arginylated residues (D and E) were defined as positive training examples. A residue not observed to be arginylated does not necessarily imply non-arginylation and therefore, a definitive set of negative examples was difficult to obtain. As an alternative, *unlabeled* data sets were constructed in two ways. First, all occurrences of D and E in proteins containing positive examples but not observed to be arginylated were included in the control (unlabeled) set. This is referred to as the *restricted*

unlabeled set because it is less likely to contain unidentified positive examples. Second, a random set of 10,000 D and E residues were sampled from all proteins in the human and mouse reference proteomes to create a *true unlabeled* set. During sampling it was ensured that the distribution of D and E residues was the same as that of the positive set (described below), known arginylation sites and sequences with non-standard amino acids such as selenocysteine were excluded. Note that both types of unlabeled sets are dependent on the positive set used during their construction.

N-terminal arginylation sites (positive examples) came from two sources. First, data from previous publications (Kurosaka et al., 2012; Leite et al., 2016; Lian et al., 2014; Saha et al., 2011; Wong et al., 2007) on arginylation were compiled into a single data set referred to as the *literature* set. These included different types of arginylation on multiple residues and only N-terminal arginylated D and E residues were considered for this study. Second, N-terminal arginylated residues identified through two different peptide array experiments in this study were added to the above data set in the following manner. For the first peptide array, a simple motif-based predictor (described below) was built using the literature data set and the restricted unlabeled set corresponding to it. This predictor was then run on the non-redundant reference proteome set, thus, scoring each occurrence of an aspartic acid or glutamic acid, based on the 10 residues C-terminal to it. From this set of 11-residue peptides, 21 were selected for synthesis (the top 16 and the bottom five scoring peptides) and assayed for arginylation. This was repeated on a set of artificially generated sequences to obtain an additional 22 synthetic peptides in a similar manner (16 top- and six bottom-scoring). This *in silico* generation involved sampling residues at each position in a peptide, randomly from position-specific distributions observed in published arginylated peptides. Of these 43 peptides, only those that were observed to be arginylated in the

assay were included in the *array 1* set. For the second peptide array, a logistic regression ensemble predictor (described below) was first trained using an updated positive set (peptides from literature and array 1) and a true unlabeled set, constructed based on this updated positive set. As before, this predictor was then run on all peptides starting with D or E from the non-redundant reference proteome set, except those at the C-terminal end to ensure that all scored peptides were 11 residues long. This predictor outputs a minimum score of zero and a maximum score of one and, therefore, selection for synthesis was performed based on three score ranges: (1) low (< 0.35), (2) medium (≥ 0.35 and < 0.65), and (3) high (≥ 0.65). Furthermore, peptides with additional D or E residues in positions other than the first position or peptides that were more than 50% identical to any other peptide were excluded. In total, 37 peptides were randomly selected in each of these three score ranges for both human and mouse, resulting in 37 x 3 x 2 = 222 peptides being synthesized and assayed. Again, only those that were observed to be arginylated in replicates were included in the data set referred to as the *array 2* set. Thus, the combined positive set that was finally used to train the predictor contained experimentally identified N-terminal arginylation sites from the literature, and array sets 1 and 2 (Table S1).

Generation of shuffled versions of known arginylated peptides. To check for positional dependence among the 10 residues C-terminal to the arginylation site, the experimental design described in (Khanal et al., 2015) was adopted with some modifications. Thirty-eight peptides were randomly selected from the literature set and the array 1 set (combined) and synthesized. Shuffled versions for each of these peptides were then generated computationally and also synthesized. During shuffling, it was ensured that the D or E in the first position remained fixed and that the shuffled sequence shared no greater than 40% identity with the original sequence. All 76 peptides were then assayed for N-terminal arginylation on the same peptide array.

II. Algorithm development

Data preparation and redundancy removal. Each positive and unlabeled site was associated with an 11-residue peptide starting with a D or E (residues that are most often arginylated) in the first position. This not only mimics the experimental setup under which the original identifications were made but also likely reflects the natural conditions for arginylation to occur: an N-terminally exposed D or E residue. To use a non-redundant training set and achieve good generalization, all residues associated with peptides that were more than 40% identical to other peptides in the data set were removed (Iakoucheva et al., 2004; Pejaver et al., 2014; Radivojac et al., 2010). In cases where a fragment containing an unlabeled site was 40% identical to a fragment containing an unlabeled site, the one with the negative class label was removed because its class designation was less reliable.

Feature extraction. Nearly all of the N-terminal arginylation sites in the training data set were identified through peptide-based experiments. Therefore, only sequence-based properties as described in previous works, were encoded in the feature matrix (Daily et al., 2005; Pejaver et al., 2014; Radivojac et al., 2010). Amino acid relative frequencies (20 frequency values) and beta entropies ($\beta \in [1, 1.25, 1.50, 1.75]$, i.e. four entropy values) (Daróczy, 1970) were calculated over multiple windows, of different lengths, C-terminal to the first position of the peptide. The net and total charge were also calculated by counting the number of positively charged residues (K and R) and negatively charged residues (D and E) within these windows (two charge values). Additionally, the proportions of aromatic residues (F, Y, and W) and the charge-hydrophobicity

ratios (Uversky et al., 2000) within these windows were also calculated (two values). For this set of features, windows of lengths 3, 7, and 11 were used (including the first positions). Finally, binary features indicating the presence (one) or absence (zero) of the 20 amino acids (plus one for non-standard residues) in each position of the peptide after the first residue were added. In total, the final feature set used in the predictor consisted of [3 x (20 + 4 + 2 + 2)] + (21 x 10) = 294 features.

Prediction models. Two models were developed to predict the propensity for a D or E residue to be arginylated: (1) a motif-based model and (2) a logistic regression ensemble model. The former served as a baseline model and the latter as the more sophisticated model that was eventually used in the final predictor.

The motif-based model utilized a naive consensus sequence-based approach to score a peptide and was “trained” as follows. A position-specific weight matrix (PWM) was calculated for the positive set by storing the frequency of each amino acid at each position in known arginylated peptides. Thus, a matrix with 21 rows (20 amino acids plus one for non-standard residues) and 11 columns (positions) was generated and was referred to as the *positive PWM*. This was repeated for the (restricted) unlabeled set and a different PWM called the *background PWM* was derived. For prediction on a new peptide, each position was iterated over and the frequency of the corresponding amino acid at a given position was extracted from the positive PWM. For example, if the frequency (probability) of finding a glycine in the third position in arginylated peptides is 0.25 and a glycine was observed in that position in the peptide to be scored, 0.25 was extracted. The probability that the given peptide sequence was generated by the PWM model of positives is simply the joint probability of seeing these 11 residues together (in the given order) in arginylated peptides. If one assumes positional independence, this probability

is the product of the 11 position-specific frequencies obtained from the positive PWM. Similarly, a probability was obtained for the background PWM as well and the positive and background probabilities were combined to obtain a single score. More formally,

$$Score = log_{10}\left(\frac{\prod_{j=1}^{11} P_{ij}^{f}}{\prod_{j=1}^{11} P_{ij}^{b}}\right)$$

where P^f and P^b are the frequencies of the observed residue at position *j*, obtained from the ith row in the positive and background PWMs, respectively (here, *i* corresponds to the row of the observed residue in the PWM). For both PWMs, any occurrence of zero was set to an extremely low value (here 10^{-10}) to prevent zero-division errors. Thus, the scores were bounded between -110 and 110. In the above expression, when the numerator is greater than the denominator, i.e. the probability for the positives is greater than that of the unlabeled, the score will be positive. Therefore, a highly positive score would suggest that the given peptide was more similar to the peptides in the positive set and a highly negative score would suggest that the peptide was more similar to those in the unlabeled set.

The second model that was trained was a logistic regression ensemble model. Logistic regression classifiers are linear classifiers that use the logistic function, applied to a linear combination of features, to calculate posterior probabilities that a data point (here, a peptide) belongs to the positive class (N-terminal arginylated). During the training process, weights associated with different features are learned in an iterative fashion so as to maximize the likelihood of the model parameters (weights), given the data. To ensure stability in training and enhance performance, the feature matrix was first Z-score normalized and the principal component analysis (PCA) was performed with the retained variance set to 95% to remove (nearly) collinear features. No further parameter optimization or feature selection was performed.

Although logistic regression models are robust to noise (typical in biological data sets), a bootstrap aggregating, or bagging, approach was used to further minimize overfitting. The training set was randomly sampled with replacement 30 times to create bootstrapped data sets (of the same size as the original training set), which were then used to train 30 logistic regression models. During training, positive and unlabeled examples were sampled separately to ensure an equal number of examples from each class in each bootstrapped data set. Thus, the final predictor utilized an ensemble of logistic regression models, each outputting scores between zero and one. These scores were then averaged to obtain the final prediction for a data point.

Evaluation. Model performance was measured in 10-fold cross-validation experiments, in which the data set was randomly divided into 10 partitions (each accounting for 10% of the data) and each partition was iteratively used as the test set, with the remaining data used for training. Partitions for cross-validation were defined at the protein level rather than the site (residue) level to avoid overestimation of performance arising from intra-protein biases. In addition, normalization and transform matrices for PCA were calculated on the training partition only and then applied to the test data. Performance measures were calculated by setting a threshold score to determine whether a D or E was predicted to be arginylated (greater than or equal to the threshold) or not (lower than the threshold). This was repeated for different score thresholds by varying them in fixed small increments. Sensitivity (*sn*; true positive rate) and specificity (*sp*; true negative rate) were then calculated at each threshold as follows:

$$sn = \frac{TP}{TP + FN}$$

$$sp = \frac{TN}{TN + FP}$$

Here, *TP* = number of true positives, i.e. instances where a positive example is predicted to be a

positive; *TN* = number of true negatives, i.e. instances where a negative (unlabeled) example is predicted to be a negative; *FP* = number of false positives, i.e. instances where a negative example is predicted to be a positive; *FN* = number of false negatives, i.e. instances where a positive example is predicted to be a negative. The receiver operating characteristic (ROC) curve was then obtained by plotting these true positive rates against the false positive rates ($fpr = 1 - sp$) at the various threshold values. The area under this curve (AUC) was calculated using the trapezoid method and was used as the main performance measure. The closer this value to one, the higher the probability that a randomly chosen arginylation site was scored higher than a randomly chosen D or E by the model.

Estimation of fractions of N-terminal arginylated residues

The logistic regression models trained in this study output a score distribution that merely approximates the true class posterior distribution. Therefore, running the predictors on the human and mouse proteomes and estimating the fraction of arginylation sites in these proteomes without enforcing a predetermined threshold (and *fpr*) is non-trivial. A more sophisticated approach was required and a previously developed protocol to derive the posterior distribution and estimate these fractions was adopted (Pejaver et al., 2017). First, a modified version of the logistic regression ensemble predictor was trained using the positive set and the true unlabeled set. Next, the class priors (fractions of positives) were estimated on the true unlabeled set by running the AlphaMax algorithm on the prediction scores from cross-validation experiments (Jain et al., December 2016). This positive-unlabeled predictor was then run on the human and mouse reference proteomes to obtain a score distribution for all D and E residues in each proteome. A transformation (described in (Jain et al., December 2016)) using the above estimates of fractions

of positives in the unlabeled set (class priors) and incorrectly labeled data points in the positive set was then applied to this score distribution to obtain the true posterior distribution on the reference proteomes. The fraction of N-terminal arginylation sites in a proteome was then estimated empirically by taking the average of the posterior probabilities for all D and E residues in the proteome. Since N-terminal arginylation requires that the site be N-terminally exposed, a more conservative estimate was derived by considering only those D and E residues in the proteome that were either in the first or second position of a protein.
